# 3D Muscle Deformation Mapping at Submaximal Isometric Contractions: Applications to Aging Muscle

**DOI:** 10.3389/fphys.2020.600590

**Published:** 2020-12-03

**Authors:** Vadim Malis, Usha Sinha, Shantanu Sinha

**Affiliations:** ^1^Department of Physics, University of California, San Diego, San Diego, CA, United States; ^2^Department of Radiology, University of California, San Diego, San Diego, CA, United States; ^3^Department of Physics, San Diego State University, San Diego, CA, United States

**Keywords:** strain and strain rate tensor mapping, aging muscle, shear strain, lateral transmission of force, compressed sensing dynamic MRI

## Abstract

3D strain or strain rate tensor mapping comprehensively captures regional muscle deformation. While compressive strain along the muscle fiber is a potential measure of the force generated, radial strains in the fiber cross-section may provide information on the material properties of the extracellular matrix. Additionally, shear strain may potentially inform on the shearing of the extracellular matrix; the latter has been hypothesized as the mechanism of lateral transmission of force. Here, we implement a novel fast MR method for velocity mapping to acquire multi-slice images at different % maximum voluntary contraction (MVC) for 3D strain mapping to explore deformation in the plantar-flexors under isometric contraction in a cohort of young and senior subjects. 3D strain rate and strain tensors were computed and eigenvalues and two invariants (maximum shear and volumetric strain) were extracted. Strain and strain rate indices (contractile and in-plane strain/strain rate, shear strain/strain rate) changed significantly with %MVC (30 and 60% MVC) and contractile and shear strain with age in the medial gastrocnemius. In the soleus, significant differences with age in contractile and shear strain were seen. Univariate regression revealed weak but significant correlation of *in-plane* and *shear strain* and *shear strain rate* indices to %MVC and correlation of *contractile* and *shear strain* indices to force. The ability to map strain tensor components provides unique insights into muscle physiology: with contractile strain providing an index of the force generated by the muscle fibers while the shear strain could potentially be a marker of lateral transmission of force.

## Introduction

Muscle strain is produced when a contraction is stimulated by action potentials transmitted by motor neurons (Frich et al., 2019). Electromyography (EMG) has been used to monitor muscle performance and is a measure of the active motor units in the neighborhood of the electrode (Winter, 2009; Frich et al., 2019). However, it is not a direct measure of muscle strain. Ultrasound (US) strain imaging has been used to measure strain and strain rate primarily in cardiac muscle (Bansal et al., 2010; Gorcsan and Tanaka, 2011; Mondillo et al., 2011; Yamada et al., 2014) though there have been several applications to skeletal muscle dynamic imaging as well (Lopata et al., 2010; Gijsbertse et al., 2017; Gao et al., 2018, 2019; Frich et al., 2019). Most ultrasound studies are performed in 2D that allows 2D strain estimation; however, some studies have extended it to bi-plane US studies where strain can be assessed in three orthogonal directions (Gijsbertse et al., 2017). A more recent study has reported 3D^*al*^ ultrasound strain imaging and shown it to be superior in identifying out-of-plane motion compared to 2D^*al*^ US strain imaging (Lopata et al., 2010). High temporal resolution and real time capabilities are advantages of ultrasound imaging and 3D^*al*^ US combined with dynamic MRI may provide complementary insights into muscle function.

Muscle contraction is anisotropic due to the mechanical characteristics of tissue as well as the preferred orientation of the muscle fibers. Thus, the full characterization of the muscle deformation requires a 3D strain or strain rate tensor estimation. However, 2D Magnetic Resonance Imaging (MRI) dynamic studies have reported that the deformation in the cross-section of the muscle fiber is highly anisotropic with the bulk of the deformation occurring in the plane containing the fibers (Malis et al., 2018; Sinha et al., 2018). The deformation in the direction orthogonal to the plane of the fibers is not significant. This would imply that a 2D strain measurement may be sufficient to characterize muscle deformation. However, this would require an image orientation such that the muscle fibers are in the imaging plane: this is not always possible especially given that fibers have complex 3D architectures. MR imaging has been employed to map regional muscle or cartilage deformation based on sequences that map voxel displacement (displacement encoded imaging) or voxel velocity [Velocity encoded phase contrast imaging (VE-PC)] or MR tagging to track voxel displacements (Spatial Modulation of Magnetization, SPAMM) (Chan et al., 2017; Malis et al., 2018; Sinha et al., 2018; Tan et al., 2018). In addition, deformation analysis of volumetric morphological images acquired in the relaxed and deformed states have been employed to study strain in the muscle (Pamuk et al., 2016) and in the cartilage (Menon et al., 2019). Velocity encoded MRI has been established as a method for strain and strain rate imaging of skeletal muscle (Malis et al., 2018; Sinha et al., 2018, 2020). The limitation of the velocity encoded MRI methods is the scan duration and the necessity for the subject to maintain consistent contractions for ∼70 cycles for imaging one slice. Recently, compressed sensing techniques combined with 2- and 3- dimensional, 3- directional velocity encoded MRI with significant savings in times have been successfully implemented to study skeletal muscle kinematics (Mazzoli et al., 2018; Malis et al., 2020). Compressed sensing has been successfully integrated with MR imaging for reducing acquisition times and is a general approach for reconstructing a signal that has been sampled at frequencies well below the Nyquist frequency (Lustig et al., 2007).

The loss of muscle force with age is well documented and this loss has been shown to be disproportionately larger than the loss of muscle mass (Goodpaster et al., 2006). Neural mechanisms have also been identified as another determinant of age-related loss of muscle force (Ballak et al., 2014). In addition to these determinants, the role of the extracellular matrix (ECM) in muscle force loss is also being increasingly recognized (Ramaswamy et al., 2011; Zhang and Gao, 2014). In young rodent muscles, at least 80% of force transmission occurs laterally through the ECM (Huijing et al., 1998), whereas in frail old muscle, the lateral transmission of force (LTF) pathway through the ECM is reduced ∼60% (Ramaswamy et al., 2011; Zhang and Gao, 2014). Computational models have identified that the potential underlying mechanism enabling LTF is the shear in the endomysium and further, predict that a thicker, stiffer ECM is less effective in supporting lateral transmission of force (Sharafi and Blemker, 2011). However, currently there are no techniques to quantify muscle shear strain *in vivo* and thus the contribution of the age-related extracellular matrix remodeling to muscle functional changes remains unexplored, especially in human subjects. Initial studies on 2D strain rate imaging using dynamic velocity encoded MRI at a single (35%) level of Maximum Voluntary Contraction (%MVC) showed the potential of these strain rate maps to identify tissue material property changes: lower strains measured in the aging cohort were potentially attributed to a stiffer extracellular matrix (Sinha et al., 2018). It should be noted that although strain and strain rate are measures of the contractile function, contractility of the muscle requires the evaluation of the stress/strain relationship. It is difficult to measure *in vivo* stress/strain relationships since the force from a single muscle cannot be measured. However, the relationship of submaximal contractions (% Maximum Voluntary Contractions, MVC) to strain can throw light on the muscle function. Further, the computation of the full 3D strain and strain rate tensor will enable the study of strains along the fiber, in the fiber cross-section as well as the maximum shear strain. These strain components will not only inform on the muscle contractile function (contractile strain) but also on the material properties of the ECM (radial expansion strain) including the presence of anisotropy in material properties (strain perpendicular to the plane of the muscle fibers) and very importantly, may be able to potentially identify an index of lateral transmission of force (maximum shear strain).

The objective of the current paper is to extract 3D strain and strain rate tensor maps in young and senior cohorts at three %MVC (30, 40, and 60% during isometric contraction) using a novel accelerated velocity encoded phase contrast imaging based on compressed sensing reconstruction to study age related differences in skeletal muscle regional deformation. An exploratory analysis of indices derived from the 3D strain and strain rate in the principal basis including principal eigenvalues, the invariants (volumetric and maximum shear strain/strain rate) is performed to identify deformation indices that are correlated to %MVC and to force.

## Materials and Methods

Nineteen subjects (11 young, 30.6 ± 8 years old, 4 female/7 male; 8 senior, 75 ± 5.3 years old, 5 female/4 male) were included in this study after written informed consent had been obtained. The criteria for inclusion was that subjects should be moderately active (self-reported) and should have had no surgical procedures performed on the lower leg. The study was carried out under the approval of the Medical Research Ethics Board of University of California, San Diego, and conformed to all standards for the use of human subjects in research as outlined in the Declaration of Helsinki on the use of human subjects in research.

Magnetic resonance imaging was performed on a 1.5 T Signa HD16 MR scanner (General Electric Medical Systems, Milwaukee, WI). The right leg which was the dominant leg for all subjects was chosen for the dynamic scan. Subjects were positioned feet first in the scanner in the supine position, with the right leg resting against foot pedal (Sinha et al., 2012). The foot pedal included an embedded optical fiber pressure transducer and the device was positioned in the center of a custom made 8-channel radiofrequency coil. During isometric contraction, the transducer detected the pressure exerted against the foot pedal and subsequently this was converted to a voltage by a spectrometer (Fiberscan, Luna Innovations, Roanoke, VA). This voltage was used to trigger the MR image acquisition using custom built software developed in LabView (version 14.0.1.4008. National Instruments Inc., Austin, TX). In addition to serving as a trigger for the MR acquisition, the pressure transducer voltage output was recorded at a sampling rate of 200 Hz and later converted into units of force (N) based on a calibration of the system using disc weights. Images were acquired during three submaximal, isometric contraction at 30, 40 and 60% MVC. The MR image acquisition was completed in approximately 26 cycles for the undersampled *k-*space acquisition (compared to the reference fully sampled scan, the compressed sensing acquisition was faster by a factor of 2 with an increase in temporal resolution by a factor of 2 due to decrease in views per segment from 4 to 2). Consistency of the contractions was ensured by providing the subject with real-time visual feedback of the actual force generated by the subject superposed on the target force curve to facilitate consistent contractions.

### Velocity Encoded Phase Contrast (VE-PC) Compressed Sensing Pulse Sequence

**FIGURE 1:**
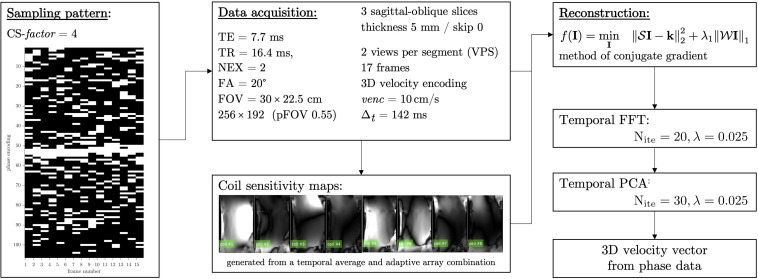
Flowchart showing the CS undersampling pattern, acquisition parameters, and the non-linear reconstruction including the sparse transforms employed here. Coil sensitivity maps derived from the multi-coil *k*-space data is integrated into the reconstruction and is shown for the eight channels of the 8-channel receive coil.

The VE-PC pulse sequence included 3 directional velocity encoding for each 2D slice. The full *k*-space VE-PC sequence was modified for random under-sampling with a different random pattern along the *k*_*y*_ direction at each temporal frame. The under-sampling along the *k*_*y*_ was a random variable density pattern with a dense sampling at the center of *k*-space. The image reconstruction used a self-calibrated coil sensitivity map generated from the data (Griswold et al., 2002) and included a two-stage non-linear iterative reconstruction using the temporal Fourier transform as the sparsifying transform in the first stage while the second stage uses the temporal PCA as the sparsifying transform (Malis et al., 2020). The regularization parameters in the reconstruction algorithm that yielded the best images (assessed qualitatively for artifacts and quantitatively for accurate velocity values compared to full *k*-space acquisition) were determined in independent optimizations performed on muscle dynamic data. The acceleration factor (CS-*factor*) was set to 4 based on the results of the previous study (Malis et al., 2020). The CS reconstruction was performed off-line using in-house developed (Malis, 2020) software developed in MATLAB (version R2020a. The MathWorks Inc. Natick, MA) running on Mac OSX (version 10.14.5 Apple Inc. Cupertino, CA). Using a computer equipped with Quad-Core Intel Core i5 CPU at 3.5 GHz with 16 GB global memory, the total computational time was ∼30 min for each series. The flow chart for the CS image acquisition and reconstruction is shown in Figure 1.

The MRI session included a localizer scan to identify the oblique sagittal orientation that best depicted the fascicles in the medial gastrocnemius. The acquisition parameters for the undersampled compressed sensing VE-PC acquisition were: TE: 7.7 ms, TR: 16.4 ms, Signal averages: 2, Flip Angle: 20°, Slice thickness: 5 mm, Field of View: 30 × 22.5 cm (partial phase Field of View: 0.55), 256 × 192 matrix (CS-*factor* of 4 combined with the lower resolution in the phase direction resulted in 26 phase encoded lines), 2 views per segment, 3 contiguous slices, 17 temporal frames (with view sharing factor = 2), 10 cm/s three directional velocity encoding. This resulted in 26 repetitions [192 (phase encode)/4 (CS-*factor*) × 0.55 (pFOV) × 2 (averages)/2 (views per segment) = 26] for each slice acquisition. The temporal resolution is calculated from: 17.8 (TR) × 2 (views per segment) × 4 (velocity encoding directions) = 142 ms. Seventeen temporal frames were collected within each isometric contraction-relaxation cycle of ∼2.4 s (17 × 142 ms = 2.4 s). Three contiguous slices were acquired at each %MVC for a total of nine dynamic acquisitions; each dynamic scan was 1 min 18 s for a total of 12 min for the complete dynamic study. It should be noted that subjects were given a short rest period (∼1 min). For each subject data collection was done in the following order 60% MVC followed by 30%, and then 40% MVC.

### Image Analysis

**FIGURE 2:**
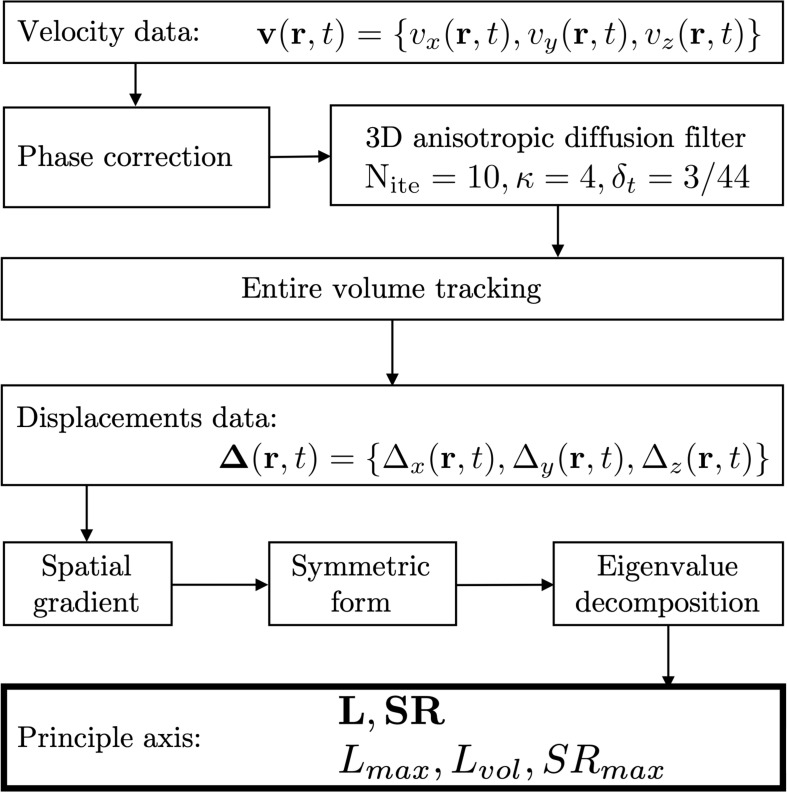
Flowchart of the image processing pipeline to estimate strain and strain rate indices. Velocity data is corrected for phase shading artifacts and denoised with a 3D anisotropic diffusion filter. Each voxel is tracked through the temporal cycle to obtain displacement maps. Spatial gradients of velocity and displacement lead to strain and strain rate maps, respectively. Lagrangian strains **L** were calculated as were the strain rates, **SR**. In addition to the eigenvalues of the tensors, two invariants were computed from the strain tensor: volumetric (*L*_*vol*_) and maximum shear strain (*L*_*max*_) and one invariant for the shear strain rate: maximum shear strain rate: *SR_*max*_.*

The flow chart for the image analysis is shown in Figure 2; the 3D tensors were calculated for the central slice of the three acquired slices. Phase images were corrected for phase shading artifacts and denoised with a 3D anisotropic diffusion filter (Perona and Malik, 1990) to yield the velocity images at the three acquired slice locations. Parameters of the anisotropic diffusion filter were: number of iterations *N* = 10, step size δ, parameter *K* controlling sensitivity to edge gradient strength = 4 and the conductance function *c*_2_ favoring wide regions over small ones, given by Eq. 1:

(1) $$c_{2}\left(||\nabla \text{I}||\right)=\frac{1}{1+{\left(||\nabla \text{I}||/K\right)}^{2}}$$

where ∇I is the gradient of the input image I. Voxels in the entire volume were tracked to obtain displacements and locations in subsequent temporal frames. The 3D strain and strain rate (SR) images were obtained by taking spatial gradient of the displacements and velocity (Eq. 2), respectively,

(2) $$\text{F}=\left[\begin{array}{ccc} \frac{\partial v_{x}}{\partial x} & \frac{\partial v_{y}}{\partial x} & \frac{\partial v_{z}}{\partial x}\\ \frac{\partial v_{x}}{\partial y} & \frac{\partial v_{y}}{\partial y} & \frac{\partial v_{z}}{\partial y}\\ \frac{\partial v_{x}}{\partial z} & \frac{\partial v_{y}}{\partial z} & \frac{\partial v_{z}}{\partial z} \end{array}\right]$$

the derived matrices were then symmetrized and diagonalized as shown for strain rate in Eq. 3, a similar computation was performed for strain. Lagrangian (**L**) strains were evaluated; Lagrangian strain uses the initial time frame as the reference length (Dandel et al., 2009). Lagrangian **SR** was computed from the velocity data.

(3) $$\mathrm{SR}=0.5\left(\text{F}+{\text{F}}^{\text{T}}\right)=\left[\begin{array}{ccc} SR_{fiber} & 0 & 0\\ 0 & SR_{out-plane} & 0\\ 0 & 0 & SR_{in-plane} \end{array}\right]$$

Once the matrices representing strain and strain rate tensors are diagonalized, the eigenvalues are sorted by ascending values. The lowest value (negative) represents compression (denoted by subscript **fiber:** the eigenvector associated with this eigenvalue is approximately along the fiber direction; at the peak of the contraction this is negative corresponding to a contraction along the fiber). The highest value (positive) represents expansion (denoted by subscript **in-plane**: component of the deformation in the fiber cross-section; at the peak of the contraction this is positive and close in magnitude to the fiber component). The intermediate eigenvalue (**out-plane**: 3rd component that is orthogonal to both **fiber** and **in-plane)** is usually very small.

Two invariants (maximum shear and volumetric strain) were calculated from the strain tensor as given by Eqs 4, 5

(4) $$L_{max}=\frac{2}{3}\sqrt{\begin{array}{cc} {\left(L_{xx}-L_{yy}\right)}^{2}+ & {\left(L_{xx}-L_{zz}\right)}^{2}+{\left(L_{yy}-L_{zz}\right)}^{2}+\\ & +6\left(L_{xy}^{2}+L_{xz}^{2}+L_{yz}^{2}\right) \end{array}}$$

(5) $$L_{vol}=\frac{\partial V}{V}=L_{xx}+L_{yy}+L_{zz}$$

and one invariant from the **SR** tensor (maximum shear strain rate, calculated similar to Eq. 4). The volumetric invariant is represented by *vol* subscript, while the maximum shear strain is represented by the *max* subscript. While the 3D tensor maps were calculated for all temporal frames in the entire volume, the values were extracted at the frame corresponding to the peak contraction and max force for strain rate and strain, respectively. Analysis was done for the values averaged in the ROIs positioned in the middle slice inside the medial gastrocnemius (MG) and soleus (Sol). The ROIs were positioned in the magnitude image of the initial frame and had a size of 7.81 × 23.44 mm as shown in Supplementary Figure S1.

### Statistical Analysis

The outcome variables of the analysis are the eigenvalues and invariants of the strain and the strain rate tensors in the principal basis. Normality of data was tested using both the Shapiro-Wilk test and by visual inspection of Q-Q plots; the strain and *SR* indices were normally distributed; data are reported as mean ± SD for all the variables. For all the indices, differences between age groups and changes with %MVCs as well as potential interaction effects (age × %MVC) were assessed using two-way factorial ANOVAs. Here, Levene’s test was used to test the assumption of homogeneity of variance and, in case of significant ANOVA results for the factor “%MVC,” Bonferroni-adjusted independent sample *t*-tests were used for *post hoc* analyses. For all tests, the level of significance was set at *p* = 0.05. Univariate linear regression was performed to identify correlation of strain and strain-rate parameters in the MG and soleus to %MVC and to force combining data from young and senior subjects. The absolute force was calculated for each subject using the 100% MVC value for the individual subject. All statistical analyses were carried out in MATLAB.

## Results

**FIGURE 3:**
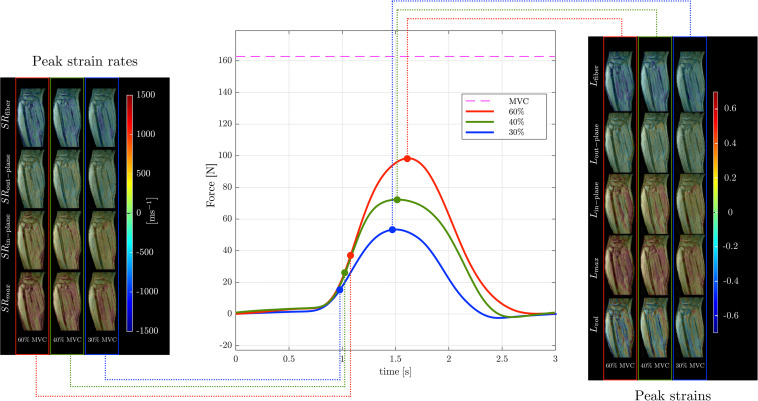
Temporal variation of forces exerted by a young subject averaged during the MR data acquisition for different force levels (center panel) along with corresponding strain (right panel) and strain rate (left panel) colormaps at the peak values (of *L*_*fiber*_ or *SR*_*fiber*_) during the contraction phase of the dynamic cycle for 60% (left column), 40% (middle column), and 30% MVC (right column). The colormap bars are shown in each panel. The temporal frames at which the peaks during contraction occurred for strain and strain rate are marked on the force curves. While the peak in strains occur at the maximum force reached, peak in strain rates occur earlier and roughly correspond to the maximum slope of the force-time curve in the contraction cycle. The subscripts, _*fiber*_, _*out*–*plane*_, _*in*–*plane*_ and *_*max*_, _*vol*_* for **SR** and **L** are explained in the text. Colormaps of the strain and strain rate indices for the entire MR acquisition cycle are presented in the Supplementary Figure S3.

**FIGURE 4:**
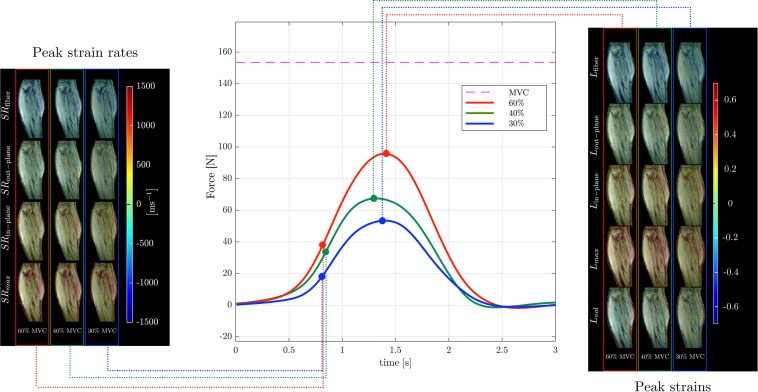
Temporal variation of forces exerted by a senior subject averaged during the MR data acquisition for different force levels (center panel) along with corresponding strain (right panel) and strain rate (left panel) colormaps at the peak values (of *L*_*fiber*_ or *SR*_*fiber*_) during the contraction phase of the dynamic cycle for 60% (left column), 40% (middle column), and 30% MVC (right column). The colormap bars are shown in each panel. The temporal frames at which the peaks during contraction occurred for strain and strain rate are marked on the force curves. While the peak in strains occur at the maximum force reached, peak in strain rates occur earlier and roughly correspond to the maximum slope of the force-time curve in the contraction cycle. The subscripts, _*fiber*_, _*out*–*plane*_, _*in*–*plane*_ and *_*max*_, _*vol*_* for **SR** and **L** are explained in the text. Colormaps of the strain and strain rate indices for the entire MR acquisition cycle are presented in Supplementary Figure S4.

Muscle force was significantly lower by 45% in the senior cohort: young (198 ± 72 N) and senior (110 ± 46 N), *p* < 0.05. All images were reconstructed using the non-linear iterative compressed sensing reconstruction following the pipeline in Figure 1. Supplementary Figure S2 shows the measurement axes with respect to the imaging plane; the longitudinal axis of the muscle is approximately along *y*. Figures 3, 4 show the maps of the eigenvalues and the maximum shear of the strain and strain rate tensors for young and senior subjects, respectively. The increase in the magnitude of deformation (eigenvalues and invariants of strain, strain rate) with %MVC in both cohorts can be visualized in these images. Further, the reduction in these parameters with age can also be visualized. The images across all the temporal frames for velocity, displacement, strain and strain rates at 30, 40, and 60% MVC are shown in Supplementary Figures S3A–L for a young subject and Supplementary Figures S4A–L for a senior subject. It should be noted that, in Supplementary Figures S3, S4, the strain or strain rate eigenvalues are indexed by λ_*1*_, λ_*2*_, and λ_*3*_ while in Figures 3, 4 the subscripts are fiber, in-plane, and out-plane. In the contraction cycle, the λ_*1*_ index corresponds to the axis approximately along the muscle fiber and is denoted with the subscript fiber; the λ_*3*_ index corresponds to the fiber cross-section direction in the imaging plane and is denoted with the subscript in-plane and the λ_*2*_ index corresponds to the fiber cross-section direction orthogonal to the imaging plane and is denoted with the subscript out-plane. Table 1 lists the values in the soleus, averaged over young and senior subjects, strain and strain rate values at the three %MVCs. Table 2 lists corresponding values in the MG. The ROI values for the deformation indices listed in Tables 1, 2 are taken at the temporal location indicated in Figures 3, 4. In accordance with the visual maps, the overall trend in all the indices is an increase (magnitude) with %MVC and a decrease with age. However, significant changes with age and with %MVC is seen in the MG while only age-related differences were primarily seen in the soleus.

**TABLE 1:** Peak strain rate and strain indices for a region of interest in the soleus averaged across young and senior subjects.

		Group	SOL		
			30% MVC	40% MVC	60% MVC
*SR*_*fiber*_	[ms^–1^]	Young	−731 ± 387	−906 ± 389	−1,102 ± 588
		Senior	^–^805 ± 683	−769 ± 344	−1,021 ± 664
*SR*_ out–*plane*_	[ms^–1^]	Young	28 ± 218	−105 ± 115	−10 ± 277
		Senior	−76 ± 85	20 ± 60	−92 ± 81
*SR*_ in–*plane*_	[ms^–1^]	Young	551 ± 196	660 ± 319	888 ± 360
		Senior	509 ± 303	530 ± 221	599 ± 321
*SR _*max*_*	[ms^–1^]	Young	1,082 ± 481	1,305 ± 516	1,656 ± 634
		Senior	1,103 ± 832	1,087 ± 410	1,363 ± 801
*L*_fiber_*		Young	−0.395 ± 0.216	−0.477 ± 0.242	−0.512 ± 0.188
		Senior	−0.287 ± 0.106	−0.327 ± 0.090	−0.363 ± 0.116
*L*_ out–*plane*_		Young	−0.016 ± 0.093	−0.053 ± 0.136	−0.023 ± 0.129
		Senior	^–^0.041 ± 0.056	−0.011 ± 0.065	0.005 ± 0.060
*L*_ in–*plane*_		Young	0.240 ± 0.062	0.291 ± 0.103	0.306 ± 0.097
		Senior	0.228 ± 0.125	0.285 ± 0.117	0.264 ± 0.116
L _max_*		Young	0.519 ± 0.212	0.630 ± 0.265	0.682 ± 0.194
		Senior	0.403 ± 0.156	0.475 ± 0.141	0.498 ± 0.172
*L _*vol*_*		Young	−0.171 ± 0.268	−0.295 ± 0.297	−0.248 ± 0.327
		Senior	−0.107 ± 0.195	−0.086 ± 0.206	−0.184 ± 0.086

*L _fiber_, L _max_, p < 0.05 between age groups.

**TABLE 2:** Peak strain rate and strain indices for a region of interest in GM averaged across young and senior subjects.

		Group	GM		
			30% MVC	40% MVC	60% MVC
*SR*_*fiber*_^†^	[ms^–1^]	Young	−596 ± 290	−777 ± 282	−940 ± 316
		Senior	−651 ± 375	−709 ± 327	−922 ± 421
*SR*_ out–*plane*_	[ms^–1^]	Young	−96 ± 174	−31 ± 137	−76 ± 91
		Senior	−71 ± 171	−49 ± 112	−47 ± 194
*SR*_ in–*plane*_^†^	[ms^–1^]	Young	583 ± 339	744 ± 270	961 ± 336
		Senior	577 ± 302	726 ± 415	945 ± 538
*SR _*max*_*^†^	[ms^–1^]	Young	992 ± 503	1,270 ± 315	1,587 ± 473
		Senior	1,025 ± 548	1,197 ± 579	1,552 ± 706
*L*_*fiber*_^†^		Young	−0.254 ± 0.129	−0.371 ± 0.176	−0.420 ± 0.120
		Senior	−0.235 ± 0.127	−0.265 ± 0.103	−0.354 ± 0.179
*L*_ out–*plane*_		Young	−0.003 ± 0.085	0.033 ± 0.128	−0.001 ± 0.142
		Senior	−0.014 ± 0.076	0.002 ± 0.058	−0.004 ± 0.109
*L*_ in–plane_*^†^		Young	0.422 ± 0.116	0.569 ± 0.161	0.697 ± 0.204
		Senior	0.378 ± 0.197	0.459 ± 0.215	0.543 ± 0.217
L _max_*^†^		Young	0.552 ± 0.175	0.765 ± 0.243	0.899 ± 0.221
		Senior	0.509 ± 0.266	0.600 ± 0.256	0.715 ± 0.297
*L _*vol*_*		Young	0.169 ± 0.212	0.305 ± 0.155	0.350 ± 0.249
		Senior	0.153 ± 0.183	0.203 ± 0.160	0.177 ± 0.253

*L_ in–plane_, L _max_ p < 0.05 between age groups. ^†^SR _fiber_, SR_ in–plane_, SR _max_, L _fiber_, L_ in–plane_, L _max_ p < 0.05 between 30 and 60% MVC.

Univariate regression of the strain and strain rate indices to %MVC for combined data from young and senior subjects identified the following indices with significant correlations: *SR _*max*_*, *SR*_*in*–*plane*_, *L _*max*_*, *SR*_*fiber*_, *L*_*fiber*_, and *L*_*in*–*plane*_ (Table 3). The indices with significant correlation to force in the combined cohort were: *L*_*fiber*_ and *L _*max*_* (Table 3).

**TABLE 3:** Univariate linear regression to %MVC and absolute force for strain and strain rate indices obtained for all the volunteers.

	%MVC			Force	
	β	*p*		β	*p*
*SR _max_*	0.36	<0.001	*L*_fiber_	−0.34	<0.001
*SR*_*in*–plane_	0.36	<0.001	*L _max_*	0.32	<0.001
*L _max_*	0.33	<0.001			
*SR*_fiber_	−0.29	0.002			
*L*_fiber_	−0.28	0.003			
*L*_ in–plane_	0.27	0.004			

## Discussion

This study reports the 3D strain and strain rate mapping of skeletal muscle at three submaximal contractions and application to monitoring differences in these indices in the calf muscle between young and senior subjects during an isometric contraction. The ability to acquire dynamic data at different %MVC was enabled by the increase in acquisition speed afforded by the in-house developed compressed sensing reconstruction integrated with randomly under-sampled velocity encoded phase contrast acquisition. An acceleration factor of 4 with an increase in temporal resolution by a factor of 2 was possible with the customized CS VE-PC sequence. This reduction in scan time allowed acquisition of 3 slices at 3% MVCs each in 12 min. An earlier study reported a randomly under-sampled 3D^*al*^, 3-directional VE-PC sequence with compressed sensing reconstruction (Mazzoli et al., 2018). However, this latter 3D^*al*^ sequence was 2 min 46 s long even for the highest achieved under-sampling factor of 4.6; consistent contractions for this length of time at 40 and 60% MVC would not be practical for senior or even junior subjects. In the current work, implementation of the fast CS VE-PC sequence enabled multi-slice, 3 directional VE-PC acquisitions of sufficiently short duration that it could be tolerated even by the senior cohort. Jensen et al. (2015, 2016) used sequential acquisition of 2D slices with velocity encoded phase contrast acquisition to compute the volumetric strain tensor under condition of passive plantarflexion. The latter papers noted that acquisition of multi-slice, 3 directional data under conditions of dynamic contractions (e.g., isometric) is more challenging than passive motion due to the requirement of a large number of consistent contractions and suggested the use of faster sequences. Karakuzu et al. (2017) performed 3D volume imaging in the “undeformed” (relaxed) and “deformed” state (sustained plantarflexion at 15% MVC) to generate strain tensors. The latter study also generated muscle fiber tracts based on diffusion tensor imaging (Karakuzu et al., 2017). The strain tensors were then rotated along the direction of muscle fiber tracts to obtain strains along muscle fascicles. The latter paper reported heterogenous strains along the muscle fascicles of the medial gastrocnemius with proximal track segments shortened, whereas distal ones were lengthened (Karakuzu et al., 2017).

There are no earlier studies of strain and strain rate imaging of the MG and soleus under isometric contraction to compare to the results of the current study. However, there are studies of other muscles and/or under different dynamic paradigms that are summarized below to offer a comparison with findings of the current paper. Strain imaging of the thigh muscle (*vastus intermedius*) of a young subject performing isometric contraction at 30% MVC using 3D ultrasound study reported strain values along the direction of contraction at −7% (Gijsbertse et al., 2017). These values are smaller than the strain values for young subjects at 30% MVC for MG (∼19%) and Sol (∼38%) reported in the current study. The differences could be related to the different muscles investigated in the two studies as well as that the 3D US study reported strain along the measurement axes rather than along the principal axes (the latter will yield bigger values). Englund et al. (2011) used spin tag MRI to compute principal strains in the anterior tibialis during 50% MVC isometric dorsiflexion contractions. Principal negative strains in the deep compartment was ∼−40% while it was ∼−24% in the superficial compartment of the anterior tibialis. The latter values compare well with the current study: i.e., to strain values at ∼50% MVC values obtained by interpolating strain values at the acquired 40 and 60% MVC values (∼−33% for MG and ∼48% for Sol). Mazzoli et al. (2018) reported 3D strain rate values in the calf muscles during active dorsiflexion to plantarflexion motion using a 3D VE-PC sequence accelerated by compressed sensing reconstruction. The negative (and positive) SR eigenvalues are somewhat higher in the latter paper (MG: −1.6 s^–1^, Sol: 1.2 s^–1^) than that reported in the current paper (at 60% MVC, MG: 0.94 s^–1^, Sol: 1.1 s^–1^). However, the contraction paradigm is active dorsiflexion to plantarflexion in the latter study which is anticipated to have a larger contraction than the isometric contraction used in the current study. Karakuzu et al. (2017) reported fiber aligned strain (sustained plantarflexion at 15% MVC) where the proximal segments shortened by 13% while distal segments lengthened by 29%. The magnitude of the strains in the latter paper are in the same range as strains reported in the current study at 30% MVC. Prior studies reported 2D strain rate values of the MG during isometric contraction (at 30% MVC) and these values are lower than that reported in the current study (Malis et al., 2018; Sinha et al., 2018). The higher values in the current study may be a reflection that the 3D tensor yields the full magnitude compared to the 2D tensor; the latter provides only the component (in the plane of the image) of the full **SR** tensor. It should be noted that it is hard to compare findings on regional deformation across studies since there is considerable variability in the techniques used in each of these studies including the MRI acquisition methodology (VE-PC, spin tags, morphological), volume vs. single slice acquisition, ROI size and location (from whole muscle volumes to regional assessment).

Absolute values of strain and strain rate indices increased with% effort and this change was approximately linear though the trend was that the slope decreased slightly with increasing %MVC. Ultrasound measurements have shown that architectural parameters like fascicle length, pennation angle, and muscle thickness change rapidly to 30% MVC, but change little at higher levels of contraction (Hodges et al., 2003; Li J. et al., 2014) while simultaneous EMG measured changed linearly with %MVC reflecting the changes in muscle activity (Hodges et al., 2003). Two ultrasound strain imaging studies have explored the relationship between %MVC and strain. A 2D biplane US speckle tracking study reported strain imaging of the *biceps brachii* at various %MVC (30, 60, 100%MVC) on 5 subjects (Lopata et al., 2010). For voluntary contraction, the latter study identified an approximately linear correlation between force and strain. However, the ultrasound studies also showed that strain at 30% was relatively high (initial slope between 0 and 30% was steep) with smaller increases seen for the higher %MVCs which potentially indicates that force and strain are not proportional. The current findings of MRI derived strain variation with %MVC are thus similar to ultrasound derived strain measures in that there is a larger increase of strain from 0 to 30% MVC with smaller increments beyond that.

The second eigenvalue of the strain and strain rate tensors has the smallest absolute value and represents the strain (or strain rate) in the fiber cross-section perpendicular to the plane of the muscle fibers. Further, in many instances, the second eigenvalue of the strain and strain rate has a negative sign indicating there is a compression along that direction. In contrast, the deformation in the fiber cross-section in the plane of the muscle fibers has a positive value indicating radial expansion during the contraction cycle. The anisotropy in the fiber cross-section (transverse anisotropy) is seen in the velocity and displacement data as well as in the strain and strain rate eigenvalues of the MG and soleus. It should be noted that even though the slice orientation did not capture the soleus fibers entirely in the image plane, the computation of the 3D strain tensor enabled the extraction of all three eigenvalues in the principal axis basis. This highlights the fact that, with multi-slice acquisition and 3D strain/strain rate computation, there is no need to image in an orientation that has the muscle fibers in the plane of the imaging slice. Since the latter orientation is not always possible for muscle fibers traversing complex paths, the flexibility of volume/multi-slice acquisition is another advantage over single slice imaging. Transverse anisotropy is seen in the MG and soleus for both young and senior cohorts and across the three %MVCs. Anisotropy of skeletal muscle deformation has been noted in an earlier study based on optical methods (Donkelaar et al., 1999). MR studies have reported the eigenvalues of the 2D strain rate tensor and inferred the strain rate in the third direction using the incompressibility of muscle; the latter strain rate was also small in magnitude compared to the strain rates in the other two directions (Malis et al., 2018; Sinha et al., 2018). MR studies that computed the 3D strain/strain rate tensor also reported deformation anisotropy in the fiber cross-section with strain and strain rate values not significantly different from zero in the direction perpendicular to the fiber plane direction (Englund et al., 2011; Mazzoli et al., 2018). The current study confirms anisotropic deformation (quantified by strain and strain rate eigenvalues) across a range of %MVCs and in young and senior cohorts in the MG and soleus muscles during isometric plantarflexion. No patterns of change in anisotropy with %MVC and/or age were identified. A computational modeling study considered the physiological implications of transverse anisotropic deformation (Hodgson et al., 2012). The latter modeling study predicted that the force generated is much higher with anisotropic deformation in the fiber cross-section than for isotropic radial expansion; the anisotropy was hypothesized to arise from constraints to deformation introduced by a specific orientation of tensile material (e.g., costameres).

In the MG, significant changes with %MVC were seen in several strain and strain rate (*L*_*fiber*_/*SR*_*fiber*_, *L*_*in*–*plane*_/*SR*_*in*–*plane*_ and *L _*max*_*/*SR _*max*_*) indices while significant age-related difference was seen only in the strain indices (*L*_*in*–*plane*_ and *L _*max*_*). The soleus showed significant differences with age in shear strain and in compressive strain (*L _*max*_* and *L*_*fiber*_). The age-related significant differences in *L*_*in*–*plane*_ and in shear strain in the MG and in shear strain in the soleus may be related to a stiffer ECM in the aging muscle (presumably from a higher collagen content in the aging muscle). Azizi et al. (2017) reported that muscles restricted from radial expansion limit longitudinal contraction and generate less mechanical work. A stiff aging ECM may limit muscle performance possibly from both a smaller longitudinal contraction due to a stiffer ECM limiting radial expansion as well as from a decrease in myofascial force transmission pathways via lateral transmission of force. Earlier reports based on the 2D strain rate tensor mapping of MG under isometric contraction identified strain rate indices along the fiber (*SR*_*fiber*_), in the cross-section (*SR*_*in*–*plane*_) and shear strain rate (*SR _*max*_*) as significantly different with age (Sinha et al., 2018). However, the current study identified, in the MG, only the strain indices as significantly different with age (*L*_*in*–*plane*_ and *L _*max*_*). The discrepant findings with the earlier 2D strain rate study may arise from differences in 2D and 3D SR tensors. Since the tissue is deforming in 3D, the current 3D tensor estimate should be a more accurate representation of the true tissue deformation. However, another factor to consider is that the inter-subject variability of the regional deformation indices is high and the choice of the subjects in the cohorts may strongly influence the presence or absence of significant differences. In attempting to interpret the different responses in the deformation indices in the MG and Sol to %MVC and with age, it should be noted that the MG is predominantly Type II, fast twitch muscles while the soleus is a slow twitch muscle with predominantly Type I fibers (Lin, 2011). Fast twitch (Type II) muscle fibers are essential for rapid muscle force production during muscle contraction. Earlier studies have shown that there was a shift in relative activation magnitude from the slow soleus to the fast gastrocnemius muscle with increasing demands of force and speed (Moritani et al., 1991). Further, EMG studies of pedaling in humans showed that the activity in the soleus increases linearly with increasing load in the low load region while the MG does not show much change in activity in this range. At higher loads, however, the activity of MG showed the largest increase in the pedaling study (Duchateau et al., 1986). In the current study, examination of the changes of strain and strain rate with %MVC, larger changes in soleus (than in the MG) is seen going from 0 to 30% MVC; e.g., 30% MVC values of strain and strain rate are consistently higher for the soleus than for the MG. In contrast to this, the MG showed the larger increase in the 30–60% MVC range as significant increases with %MVC are only seen in the MG and not in the soleus. These findings support the EMG studies on the relative activation of slow twitch soleus at low loads that switches to the predominantly fast twitch MG at higher loads. Aging related atrophy is also muscle fiber type specific with Type II muscle fiber atrophy being more pronounced than muscles with Type I muscle fiber type. Animal and human studies have shown that atrophy in the MG muscle is much more pronounced than in the soleus in keeping with the predominant fiber type in each muscle (Picard et al., 2011; Kramer et al., 2017). However, contrary to the atrophy- based expectations that the soleus experiences less atrophy than the medial gastrocnemius, the strain tensor results showed that both the soleus and the medial gastrocnemius had significant decreases in these values with age. It is possible that muscle fiber size alone may not be the determinant of strain and strain rate magnitude. Structural studies of the calf muscles in human subjects have reported that increases in connective and adipose tissue occur with age in all the plantar flexor muscles (Csapo et al., 2014). However, larger increases in adipose tissue (fat infiltration) with age is seen in the soleus muscle than in the medial gastrocnemius muscle (Csapo et al., 2014). It is possible that the observed age-related changes in the MG and in the soleus reflect the combined effects of muscle fiber atrophy as well as non-contractile tissue infiltration. It should be noted that there was no MVC × age interaction. It was anticipated that the difference in deformation values between the young and senior cohort will be larger at 60% MVC. While this was true, this difference did not reach significance for any of the deformation parameters for either the soleus or MG.

Two invariants of the strain and strain rate tensor were calculated in this study. One of them was the shear strain (and shear strain rate). Shear strain and shear strain rate indices (latter only for the MG) were consistently identified as either significantly increased with %MVC or significantly decreased with age. 2D strain rate studies published earlier revealed shear strain rate to be significantly different between age groups and further, emerged as a significant predictor of force loss with age or with disuse atrophy (Malis et al., 2018; Sinha et al., 2018). The physiological implication for shear strain (and shear strain rate) can be inferred from computational modeling studies. Sharafi and Blemker (2011) employed computational models to predict that the shearing of the endomysium is the most likely pathway for transmission of force generated by non-spanning muscle fiber that are not attached to the tendon. This is a lateral pathway for transmission of force as opposed to the myotendinous pathway. The observed significant increase in shear strain and shear strain rate with %MVC in the MG may imply an increase in the amount of force transmitted by lateral pathways at higher %MVCs. Further, the observed significant decrease in shear strain with age in the soleus and in the MG may translate to a reduction in lateral transmission of force with age. This reduction in lateral transmission of force may then account for the loss in total force with age. The lateral transmission of force via the shearing of the endomysium discussed here is based on a simplified model of a single muscle fiber and its surrounding endomysium. The *in vivo* situation is more complex and includes groups of active muscle and in reality, the shearing of the endomysium may potentially arise from complex intramuscular myofascial loads. The observed heterogeneous spatial strain patterns in muscle have been attributed to epimuscular myofascial loads and intramuscular ones originating from the ECM and muscle fibers that influence local deformations (Yucesoy, 2010). Further, it should be noted that lateral transmission of force by the ECM is part of a more general force transmission mechanism: ‘myofascial force transmission’. The latter mechanism considers the skeletal muscle within a myofascial continuity, where ECM mechanically interacts with muscle fibers along their full lengths (Yucesoy, 2010). Recent studies have shown that these mechanical interactions have significant effects on the mechanical properties of the connective tissue (Wilke et al., 2018). Age induced structural remodeling of the ECM may alter the mechanical properties of the connective tissue resulting in differences in strain and strain rate components between young and senior cohorts.

The other invariant computed from the strain tensors is the volumetric strain which is the fractional change in volume. In a nearly incompressible tissue like muscle, this index should be close to zero. Compared to shear and shear strain eigenvalues, the volumetric strain is an order of magnitude smaller but not zero. The volumetric strain values of the anterior tibialis under passive plantarflexion in humans has been reported (Jensen et al., 2015, 2016); the latter studies revealed predominantly positive volumetric strains with a heterogeneous distribution under passive stretch. Jensen et al. reported mean values of 0.018 ± 0.026 mm^3^/mm^3^ in the most superior muscle slice and 0.26 ± 0.13 mm^3^/mm^3^ in the most medial, inferior region (Jensen et al., 2016). In comparison, the volumetric strain in the soleus is positive and negative in the gastrocnemius, while the magnitude (taken in the mid-belly of each muscle) is in the range of 0.09–0.36 mm^3^/mm^3^ in the current study. The values are comparable though it should be noted that Jensen et al. (2016) reported strains under passive stretching while the current paper is deformation mapping during active isometric contraction.

Using data for the rat spinotrapezius muscle, a computational study estimated that volumetric changes occurred during contraction and stretch as a consequence of blood and lymphatic vessels within the endomysial and perimysal spaces that change diameter and length (Causey et al., 2012). Blood volume changes in the perimysial space was shown to be primarily responsible for the deviation from isovolumetry resulting in a 7% increase with contraction and a 6% decrease with stretching. The current observation of positive volumetric strain at peak contraction in the MG (∼10%) is in conformance with the predictions of the computational study. However, the negative volumetric strain in the soleus at peak contraction (∼15% in young subjects) is difficult to explain based on the blood influx model. Calf muscle perfusion studies on human subjects after exercise may also provide information to interpret volumetric strains found in the current study. Perfusion values in the soleus post-exercise (supine plantar flexion) was lowest of all the calf muscles and lower than in the medial gastrocnemius (Vankana et al., 1998). This lower blood flow in the soleus may imply that the blood vessel diameter does not increase as much as other muscles and thus the anticipated increase in volume is not seen. However, it still does not explain the fairly high decrease in volume (∼15%) at peak contraction in the soleus. Jensen et al. (2016) also reported positive volumetric strain values in the anterior tibialis (stretch) during passive plantarflexion; this is also in contradiction to the predictions of the computational model of a volume decrease under stretch conditions. An explanation advanced for this discrepancy is that while the model predicted volume changes at the fiber/fascicle level, the MR measurements do not have the resolution to measure at this scale. The voxel level measurements of volumetric strain may have contributions from other larger scale strains.

Univariate regression analysis showed that contractile strain and shear strain correlated to force. Correlation of strain to force is promising as it indicates the possibility of using strain as a measure of muscle function and even potentially as a marker of individual muscle force. The role of shear in the endomysium as a potential mechanism of lateral transmission of force was discussed earlier. While the resolution of MRI does not allow one to directly image the endomysium or its deformation, it is reasonable to extrapolate that the measured shear strain may reflect the shear of the endomysium. Thus, the correlation of shear strain to force may potentially provide a marker of force transmission through lateral pathways. Shear strain rate was also identified as a predictor of force in a cohort of young and senior subjects in earlier studies using 2D strain rate analysis (11). In summary, while the contractile strain is a measure of the force generated by the muscle fibers; shear strain may potentially reflect the effectiveness of transfer of this force by myofascial pathways of this force to the tendons.

In the univariate regression analysis of deformation indices to %MVC, shear strain, shear strain rate as well in-plane deformation indices emerged as the top correlates to %MVC. The %MVC is a relative measure and does not take the absolute force into account. This may potentially explain why contractile strain does not show the highest correlation to %MVC. Thus, %MVC may be dependent on other factors (other than the contraction strain that is a measure of the force generated) that influence the measured force. Based on computational modeling papers as well as on physical models, it can be inferred that both shear and in-plane deformation are controlled by the material properties of the ECM (Sharafi and Blemker, 2011; Azizi et al., 2017). The age-related increase in endomysial thickness and in the amount of collagen, among others, can alter the structural properties of the ECM. This may lead to decreases in shear strain and in-plane deformation that potentially limit muscle force with age.

The current study has a few limitations: while multiple slices were acquired, a true 3D acquisition would ensure no mismatch in adjacent slices and allow a higher SNR. However, the drawback of even the compressed-sensing based 3D VE-PC sequence was its longer acquisitions times. Future work will focus on making faster 3D flow sequences incorporating e.g., flow encode randomization (Hutter et al., 2015). There is also fairly high inter-subject variability within each cohort presumably reflecting physiological variability. While the current study controlled for age and activity, more well-defined and objective criteria for subject selection may have reduced this variability. It is also likely that more pronounced differences between the two cohorts may be seen if the senior age group were above 80 years old, as there is evidence, from animal studies, of loss for force transmission pathways in very old rather than in old mice (Ramaswamy et al., 2011).

In conclusion, the current paper establishes a non-invasive method to explore regional deformations in the contracting muscle with the ability to quantify contractile strain along the fiber, radial expansion and anisotropic deformation in the fiber cross-section, and maximum shear strain. Measurements of these indices have the potential to distinguish between force production due to contraction along the fiber and lateral force transmission through shear in the endomysium. This study was enabled by the in-house developed faster compressed sensing based acquisition technique for velocity imaging of skeletal muscle to extract the full 3D strain and strain rate. The current paper is focused on the aging muscle and thus the proposed method has immediate clinical application in studying sarcopenia. Other applications of 3D strain and strain rate imaging include muscular dystrophies to study force-strain patterns (Kim et al., 2010). Further, contractile strain and shear strain mapping will provide insight into functional differences between normal and dystrophied muscle. Comparing the temporal variation of strain to the force pattern may be a sensitive method to study delayed relaxation that occurs in conditions such as myotonic disorders (Esposito et al., 2015). The method presented here includes spatial mapping of the strain and strain rate tensors and is thus ideally suited to detect focal muscle involvement, such as inflammatory muscle diseases (Walker, 2008). While the focus in the current paper is on MR deformation imaging, it should be emphasized that MR provides an arsenal of structural and functional tools including diffusion tensor imaging (Noseworthy et al., 2010) perfusion imaging (Adelnia et al., 2019), fat quantification (Fishbein et al., 2018), relaxometry (Li K.et al., 2014) and spectroscopic (Choi et al., 2016) methods for a comprehensive assessment of the normal and aging muscle.

## Data Availability Statement

The raw data supporting the conclusions of this article will be made available by the authors, without undue reservation.

## Ethics Statement

The studies involving human participants were reviewed and approved by the Medical Research Ethics Board of University of California, San Diego. The patients/participants provided their written informed consent to participate in this study.

## Author Contributions

VM performed the research and analyzed the data. All authors designed the research and wrote the manuscript.

## Conflict of Interest

The authors declare that the research was conducted in the absence of any commercial or financial relationships that could be construed as a potential conflict of interest.
