# Supplementary material for: 3D Muscle Deformation Mapping at Submaximal Isometric Contractions: Applications to Aging Muscle
Source: Front Physiol. 2020 Dec 3;11:600590. doi: 10.3389/fphys.2020.600590 (PMC7744822; doi:10.3389/fphys.2020.600590)
Supplement: Supplementary file 1 [file Data_Sheet_1.PDF]

Supplementary Material

1 Supplementary Figures

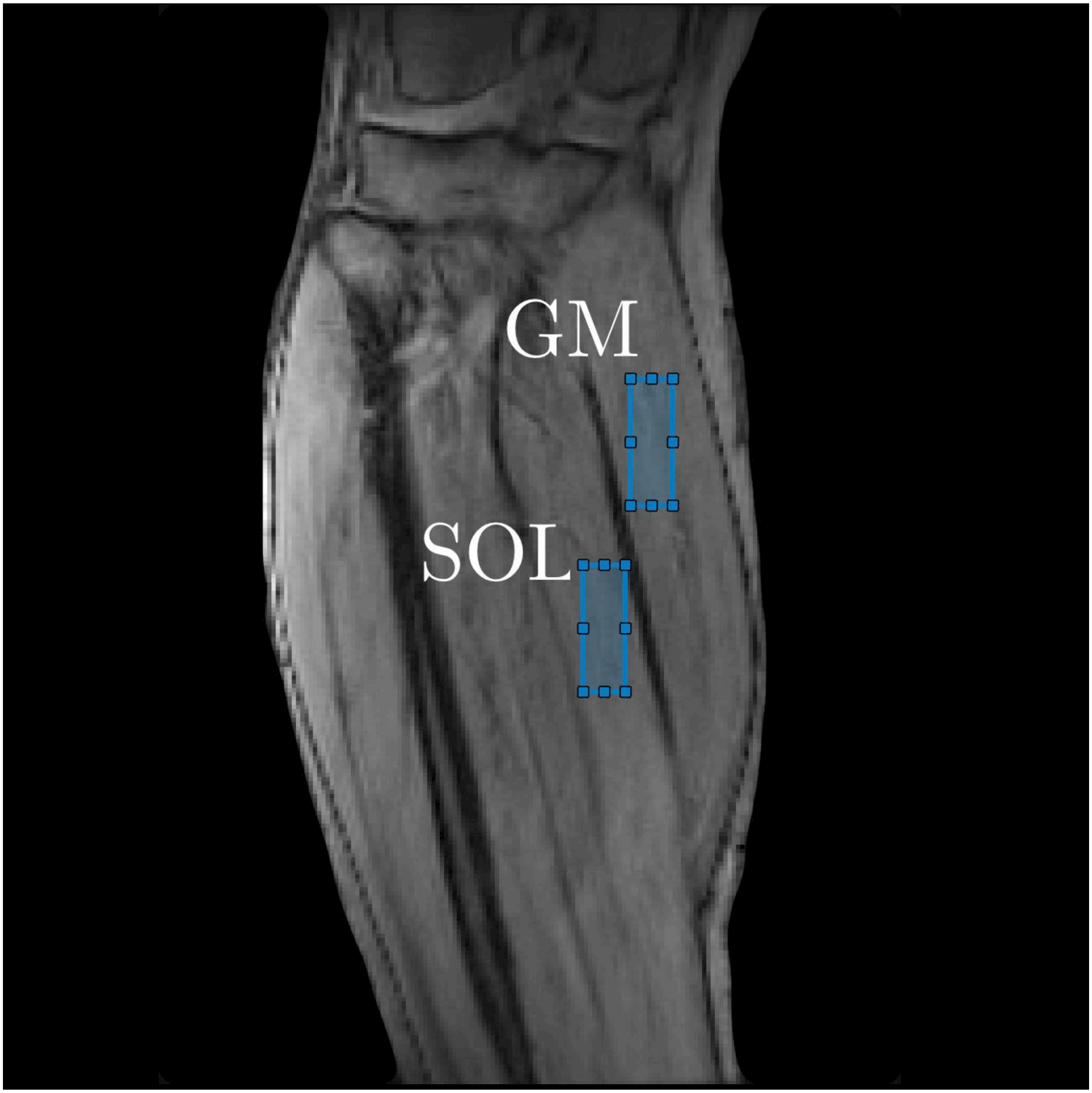

Supplementary Figure 1. Magnitude image of the first frame of the dynamic series. Strain and strain rate tensor component values were taken in the regions of interest placed in the soleus and in the medial gastrocnemius.

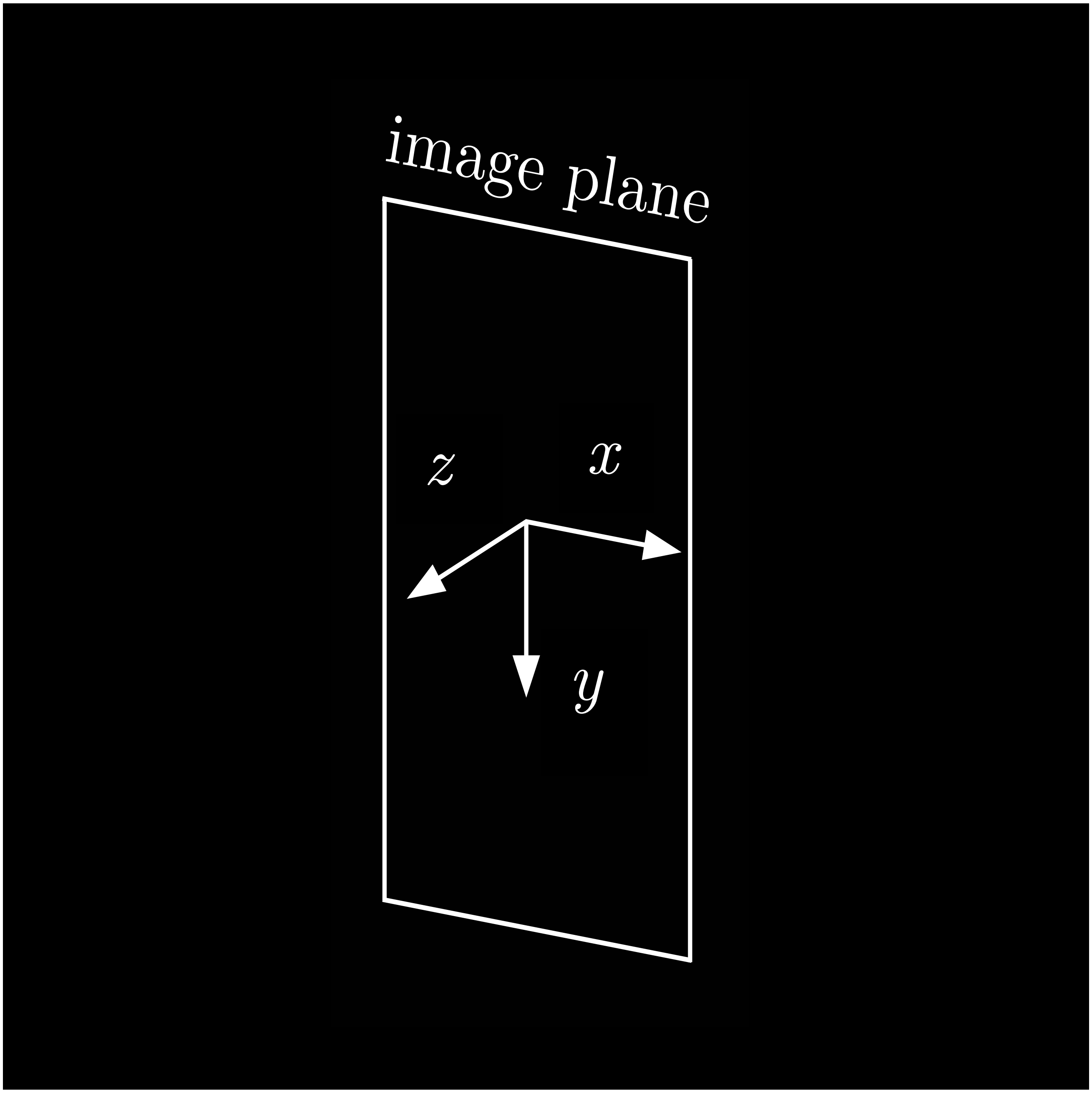

Supplementary Figure 2. The directions for the velocity and displacement vectors are shown: *x*-*y* is in the plane of the acquired image, with *y* approximately along the long axis the calf muscle and *x* is the anterior posterior direction. The *z* direction is perpendicular to the imaging plane and is in the medial-lateral direction.

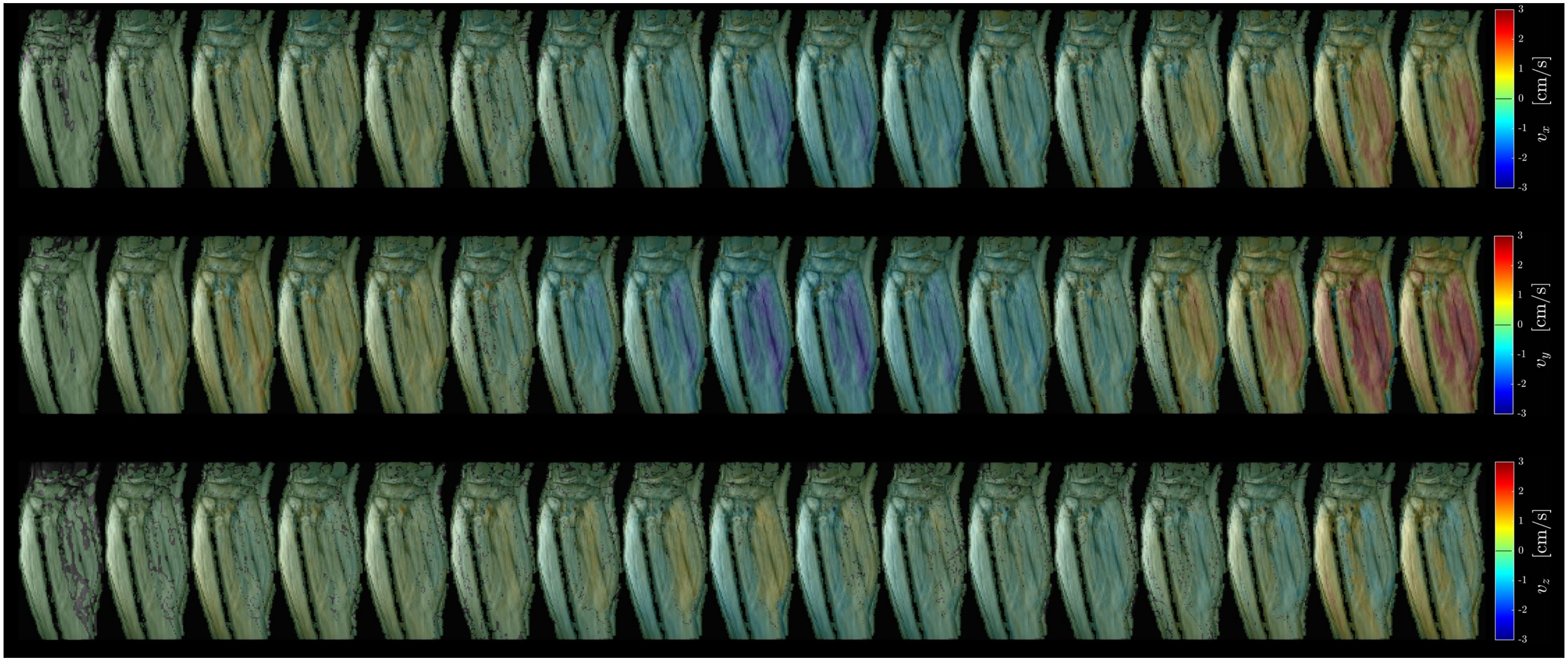

Supplementary Figure 3a. Velocity maps computed for a young subject at 60%MVC. Velocity maps are shown through the first 2.3 seconds of the 3 second temporal cycle of the isometric contraction (17 temporal frames). The transition from contraction to relaxation part of the isometric contraction occurs around temporal frame 13. The change in velocities from negative (during the contraction) to positive (during relaxation) can be visually appreciated. The velocity maps have the highest values along the *y*-axis (v_y) (longitudinal muscle axis); the muscle fiber also primarily runs in craniocaudal direction (middle row). The intermediate values are seen along the *x*-direction (v_x) which is in the imaging plane, the plane of the muscle fibers (top row). The smallest values are seen in the *z*-directions (v_z) which is the out-of-plane direction (bottom row). In addition to the temporal variations, the decrease in velocity with %MVC (comparing S3a to S3b to S3c) is also visually evident.

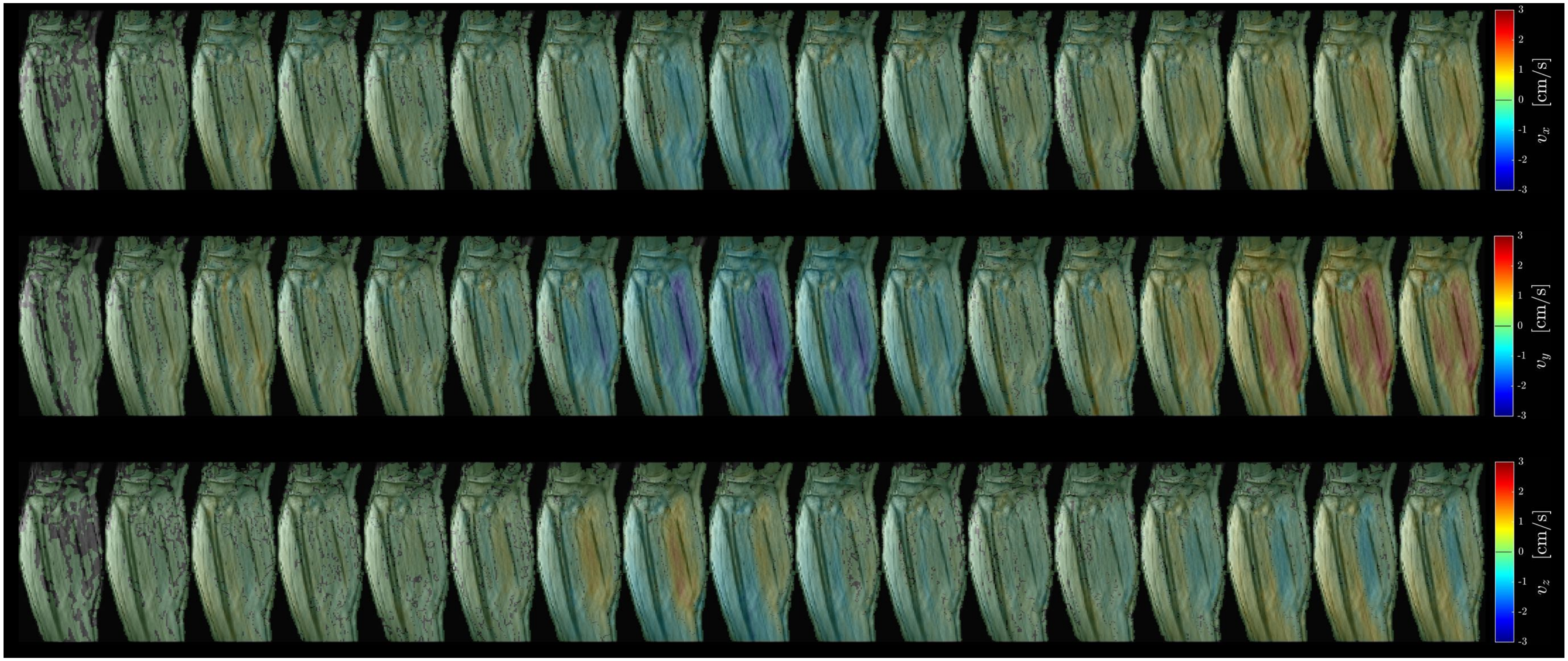

Supplementary Figure 3b. Velocity maps computed for a young subject at 40%MVC.

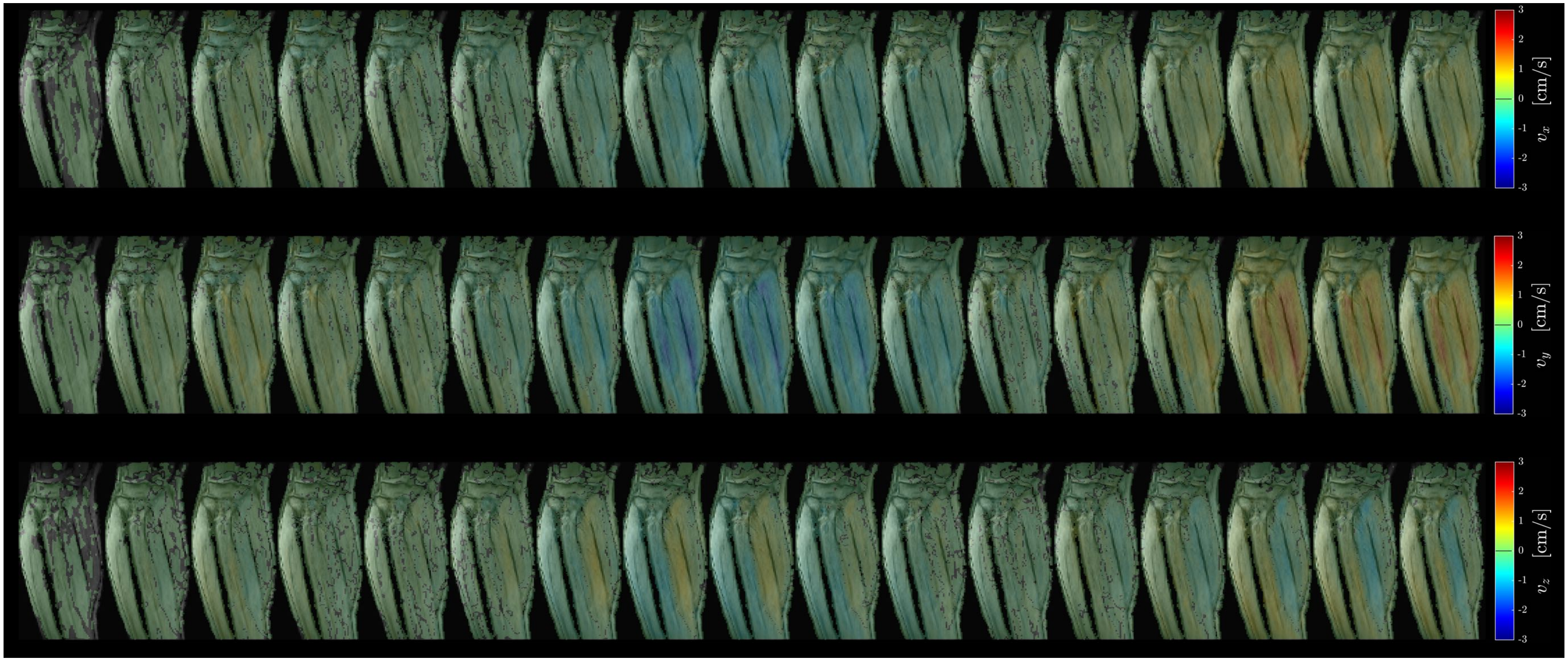

Supplementary Figure 3c. Velocity maps computed for a young subject at 30%MVC.

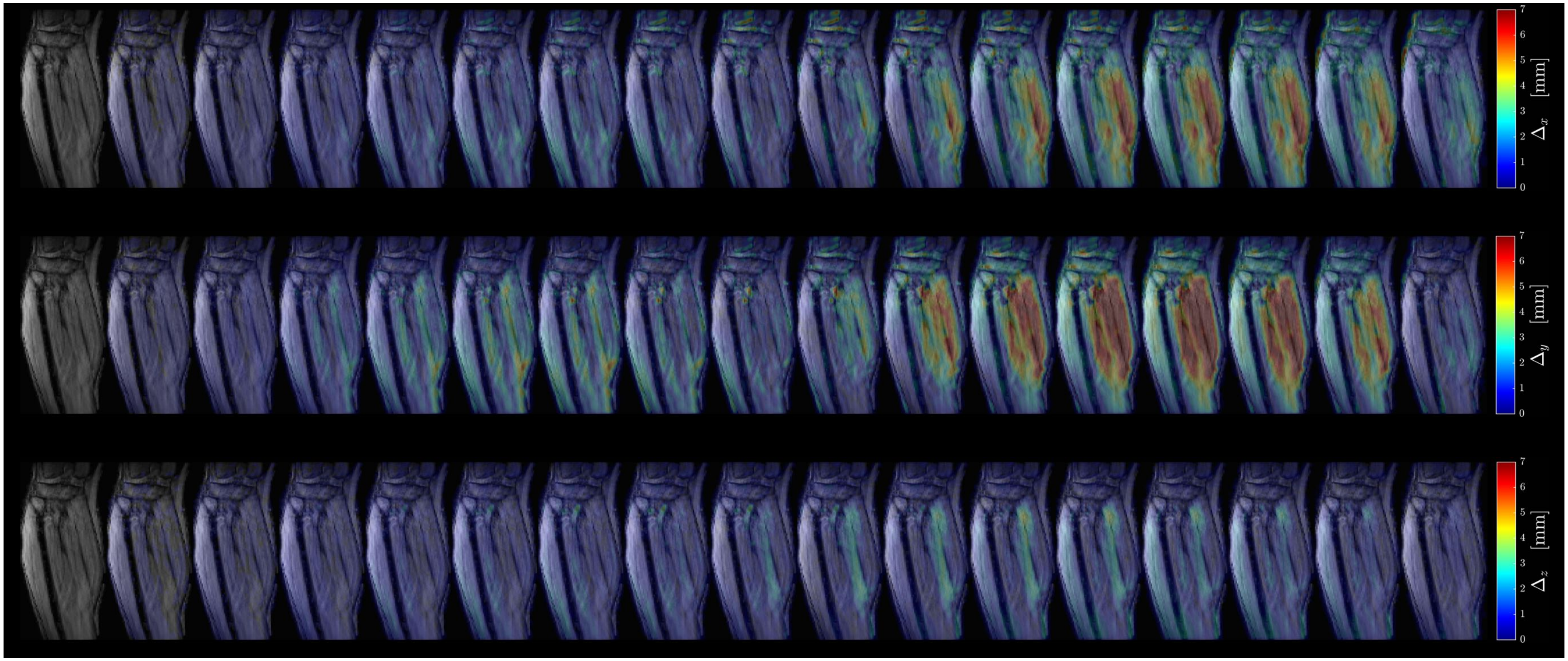

Supplementary Figure 3d. Displacement maps computed for a young subject at 60%MVC. Displacement maps are shown through the first 2.3 seconds of the 3 second temporal cycle of the isometric contraction (17 temporal frames). The maximum displacement occurs at the peak of the force curve (frame 13). The displacement maps have the highest values along the y-axis (Δ_y) in conformance with the direction of the largest velocity values. The intermediate displacement values are seen along the *x*-direction (Δ_x) which is in the imaging plane, the plane of the muscle fibers (top row). The smallest displacements are seen in the *z*-direction (Δ_z) which is the out-of-plane direction (bottom row). Displacements, similar to velocity, decrease as the MVC decreases from 60% to 30% MVC.

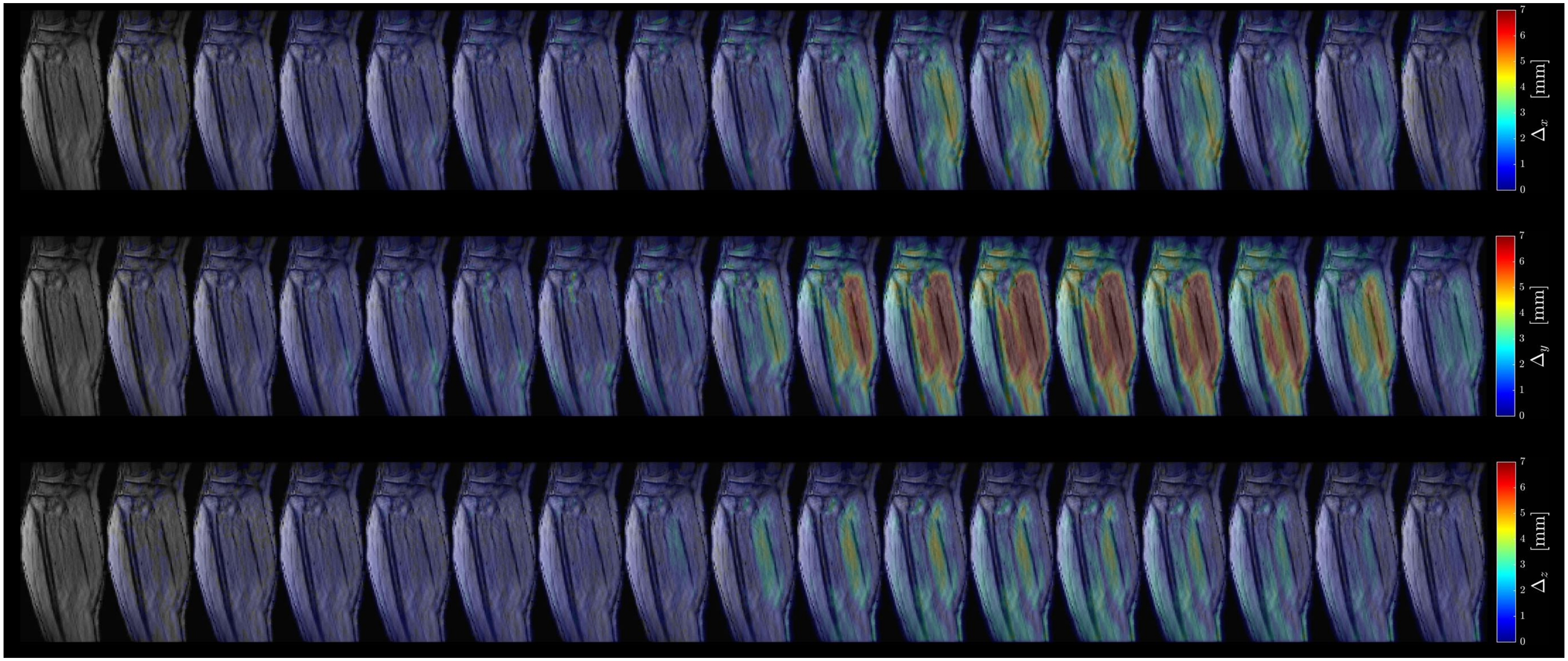

Supplementary Figure 3e. Displacement maps computed for a young subject at 40%MVC.

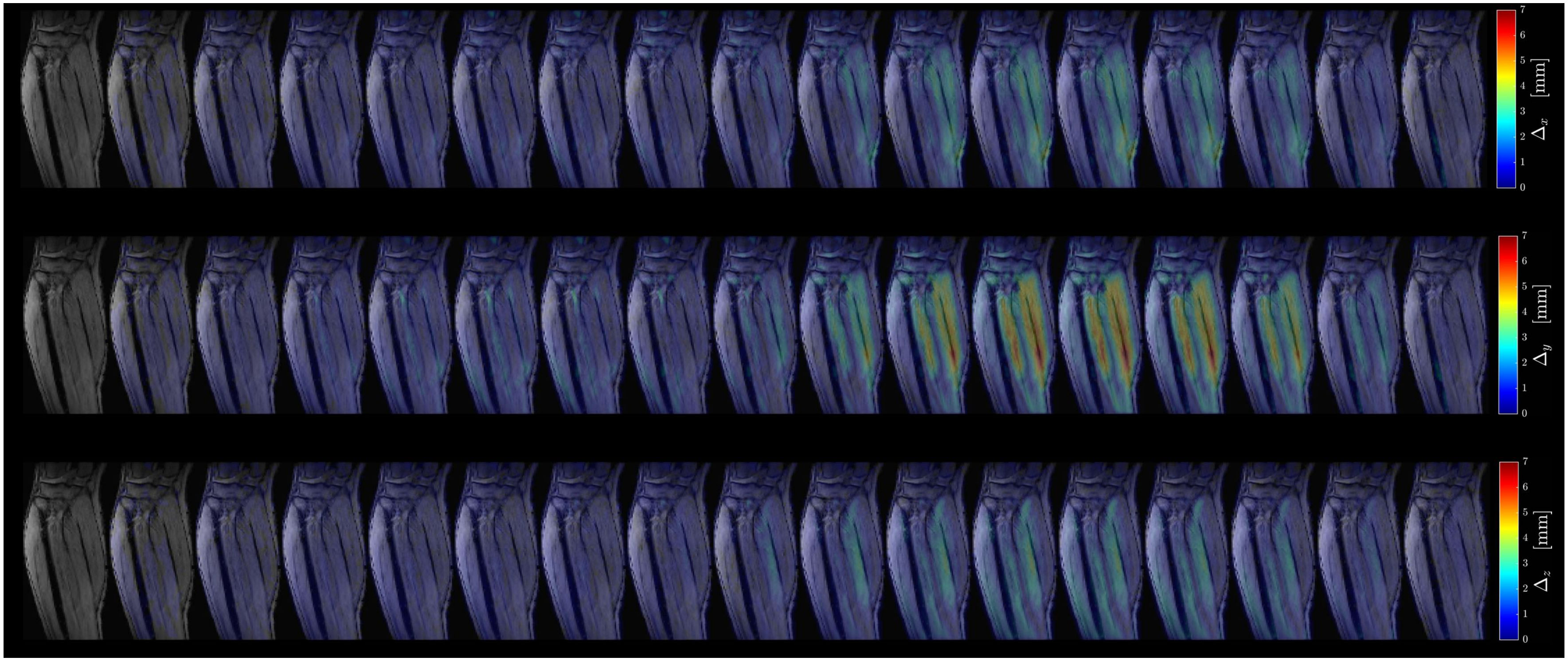

Supplementary Figure 3f. Displacement maps computed for a young subject at 30%MVC.

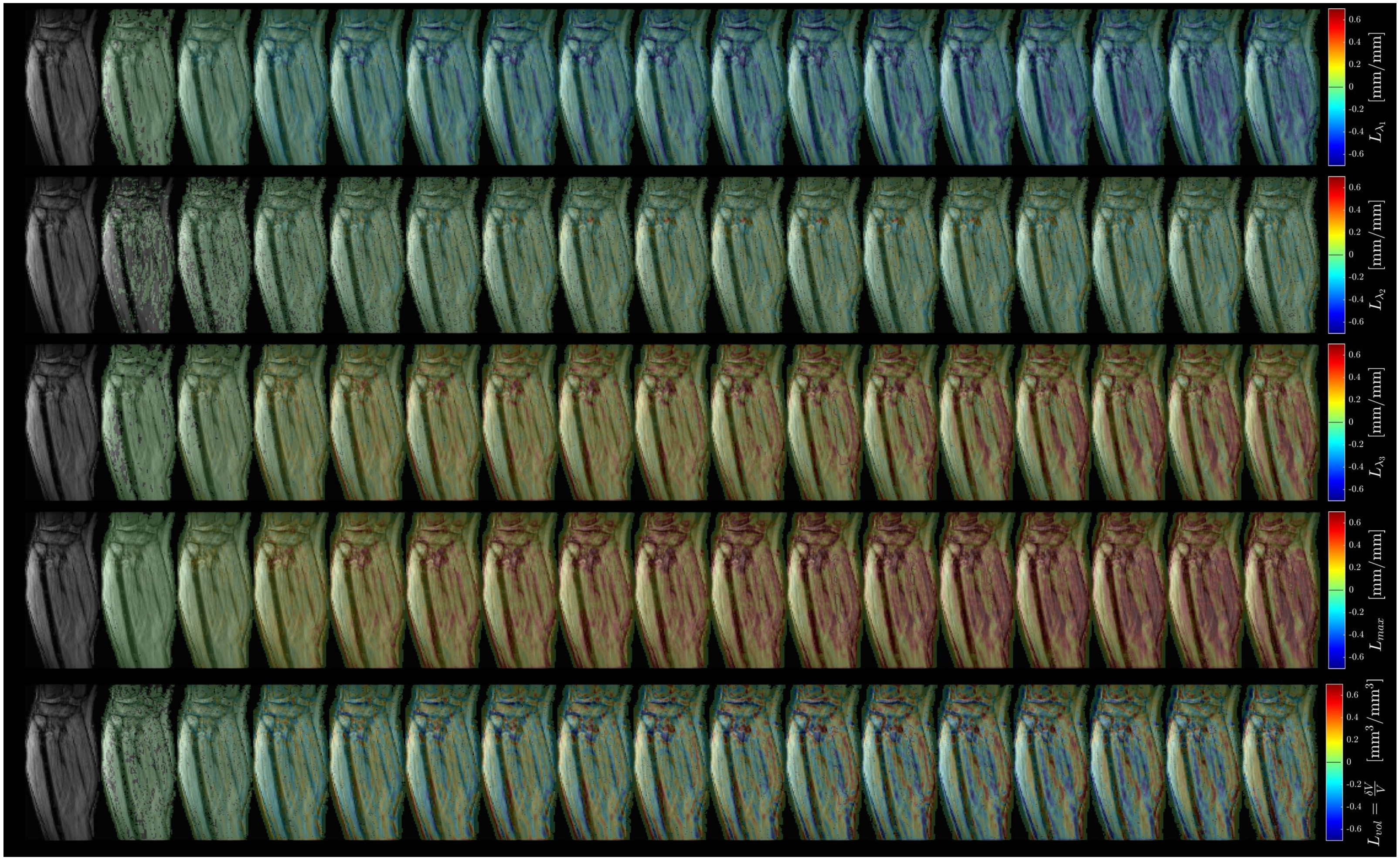

Supplementary Figure 3g. Strain maps computed for a young subject at 60%MVC. The top three rows are the eigenvalues of the strain tensor arranged in increasing values from first to the third row. The negative eigenvalue (E_{λ_1}) is shown in the top row and during the contraction phase the negative eigenvalue is approximately along the muscle fiber and denoted as E_{fiber}. The second row shows the maps of the second eigenvalue (E_{λ_2}), it has the smallest absolute value and the direction of the strain is orthogonal to the imaging plane: $E_{\text{out-plane.}}$ The third row is the map of the third eigenvalue (E_{λ_3}), it is positive, and during the contraction phase, the direction of the strain is in the fiber cross-section in the imaging plane: $E_{\text{in-plane}}$. The last two rows are the two invariants of the strain tensor: maximum shear strain: E_{max} and volumetric strain: E_{vol}. The decrease in the values of the strain components as %MVC decreases can be visually appreciated.

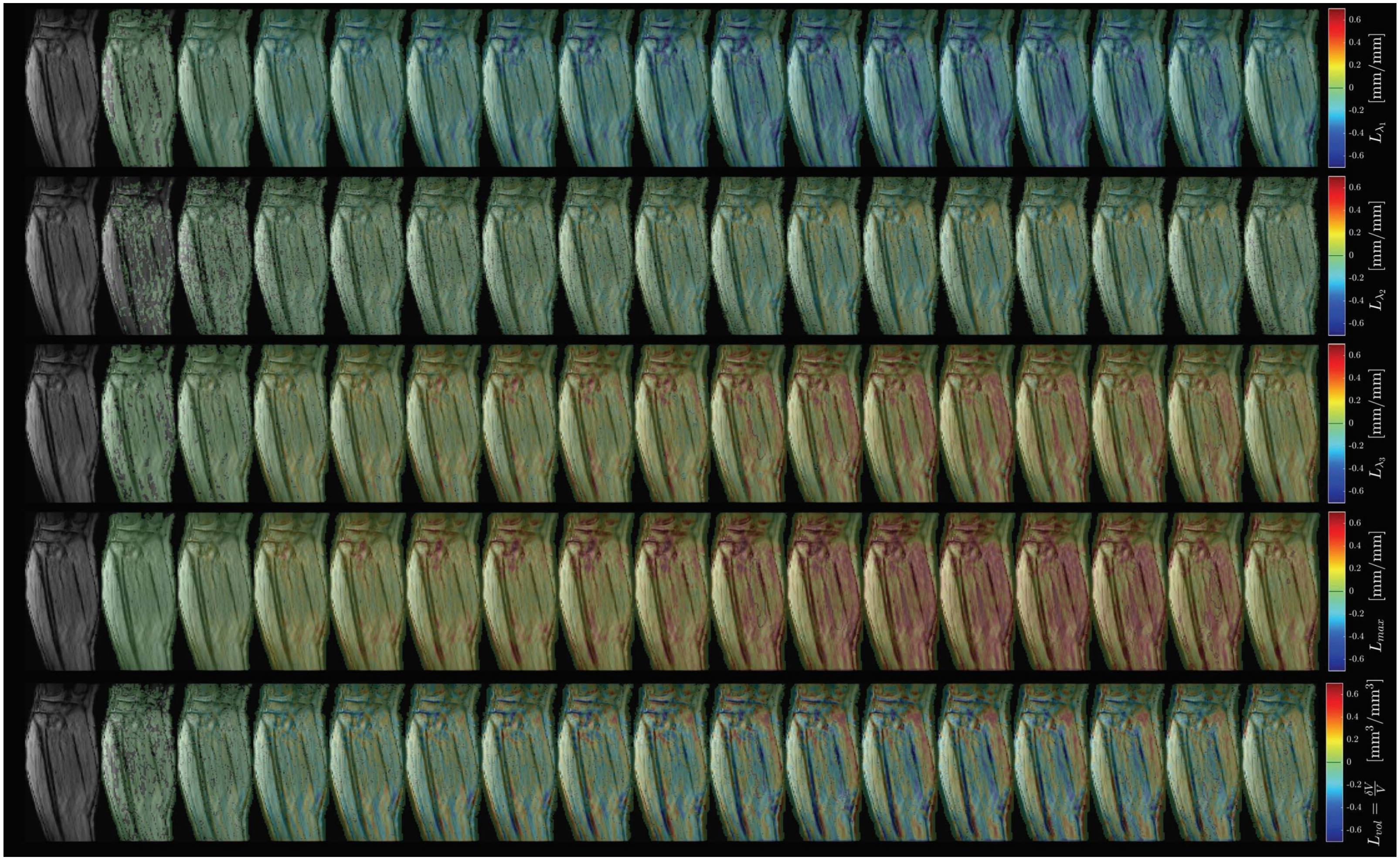

Supplementary Figure 3h. Strain maps computed for a young subject at 40%MVC.

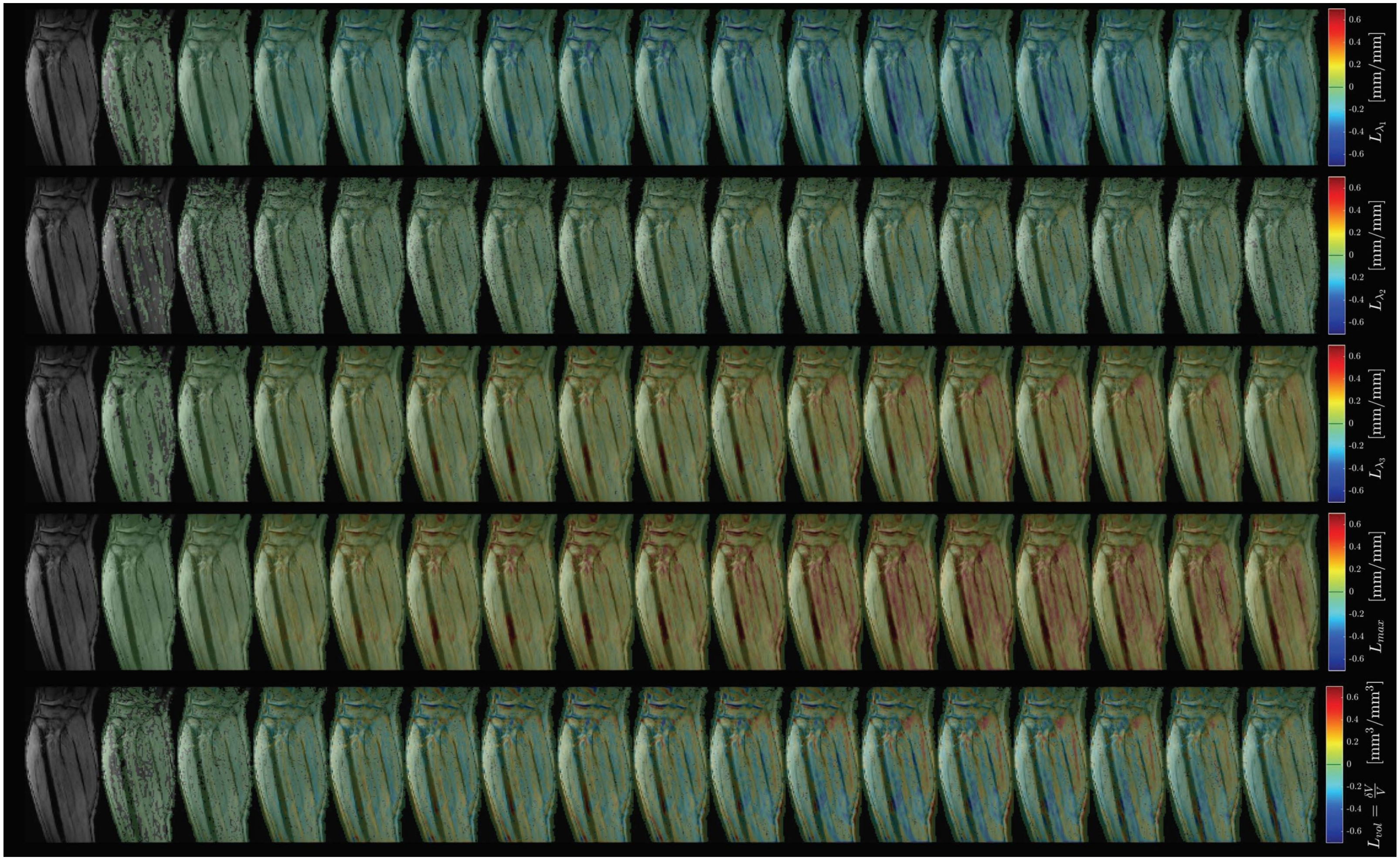

Supplementary Figure 3i. Strain maps computed for a young subject at 30%MVC.

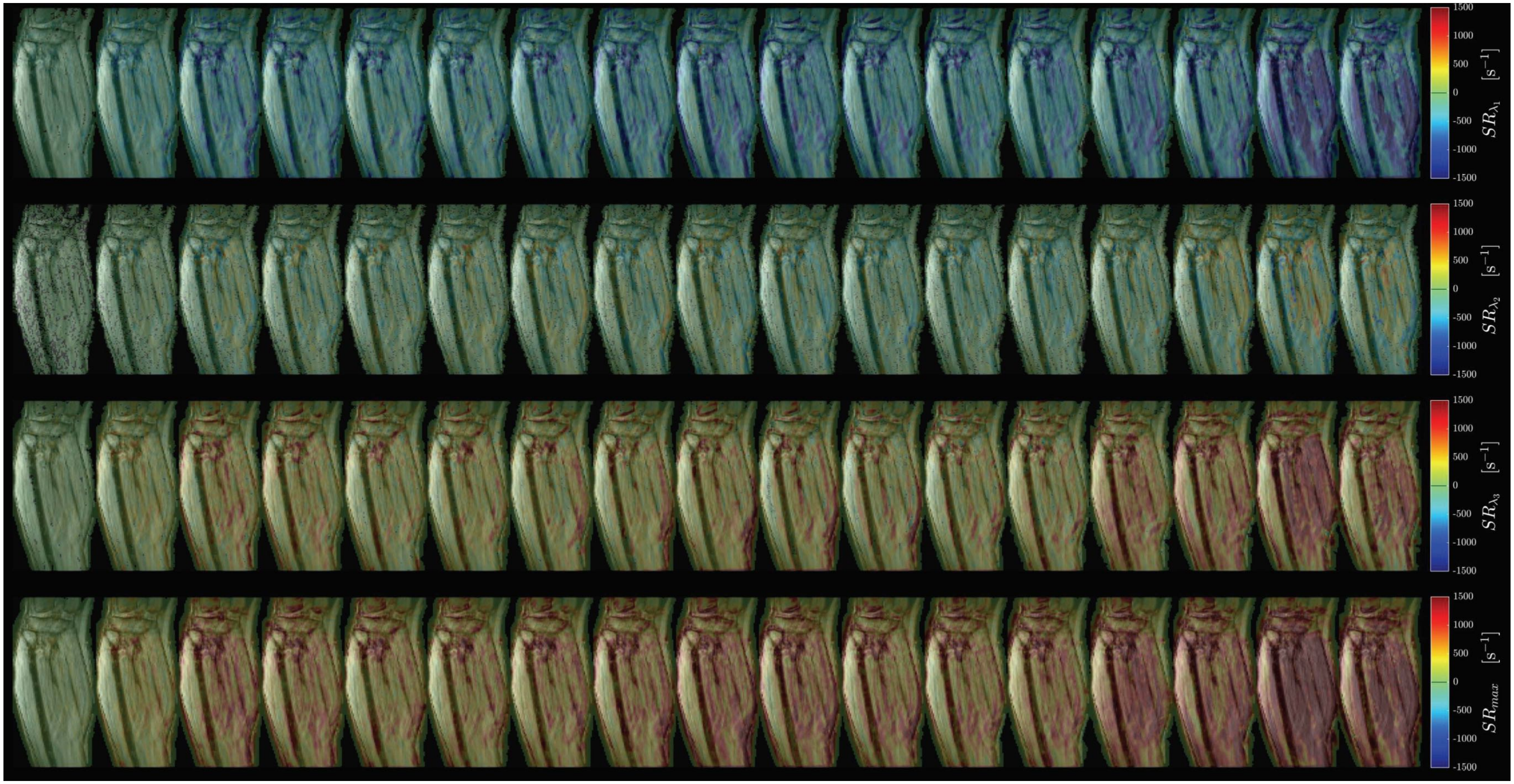

Supplementary Figure 3j. Strain rate maps computed for a young subject at 60%MVC. The top three rows are the eigenvalues of the strain rate tensor arranged in increasing values from first to the third row. The negative eigenvalue (SR_{λ_1}) is shown in the top row and during the contraction phase the negative eigenvalue is approximately along the muscle fiber and denoted as $SR_{\text{fiber.}}$ The second row shows the maps of the second eigenvalue (SR_{λ_2}), it has the smallest absolute value and the direction of the strain is orthogonal to the imaging plane: $SR_{\text{out-plane.}}$ The third row is the map of the third eigenvalue (SR_{λ_3}), it is positive, and during the contraction phase, the direction of the strain is in the fiber cross-section in the imaging plane: $SR_{\text{in-plane.}}$ The last row is the map of the maximum shear strain: SR_{max}. The decrease in the values of the strain components as %MVC decreases can be visually appreciated.

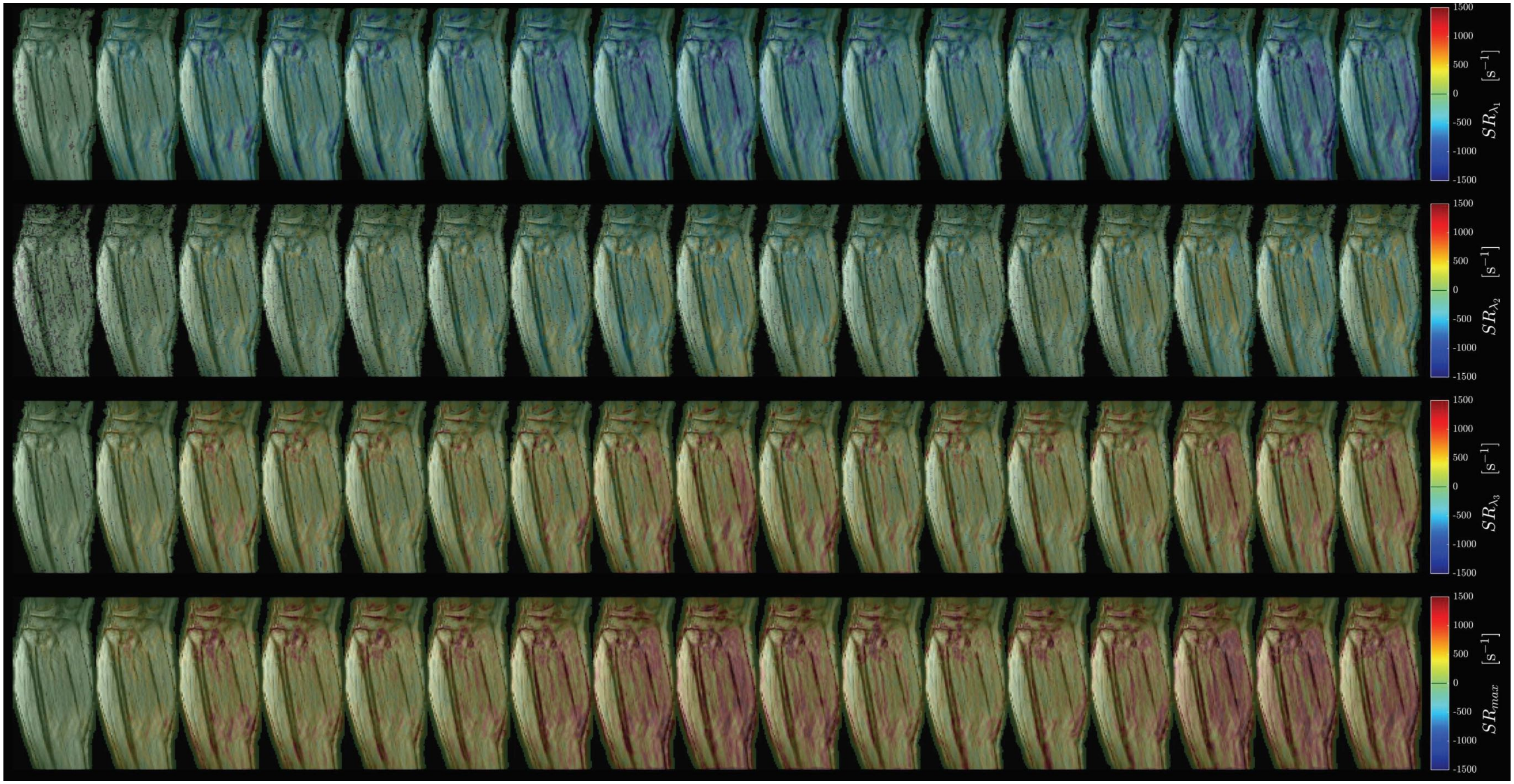

Supplementary Figure 3k. Strain rate maps computed for a young subject at 40%MVC.

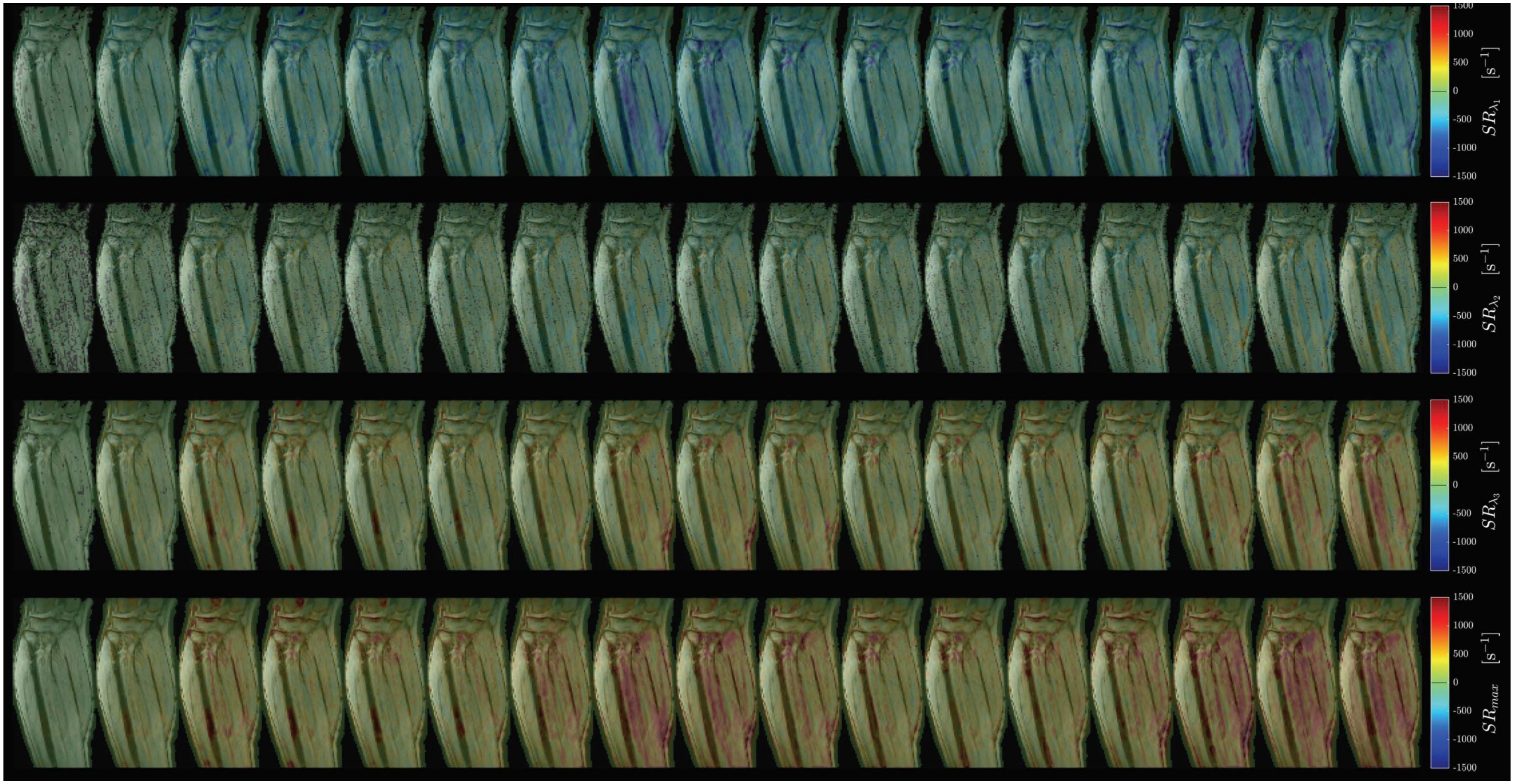

Supplementary Figure 3l. Strain rate maps computed for a young subject at 30%MVC.

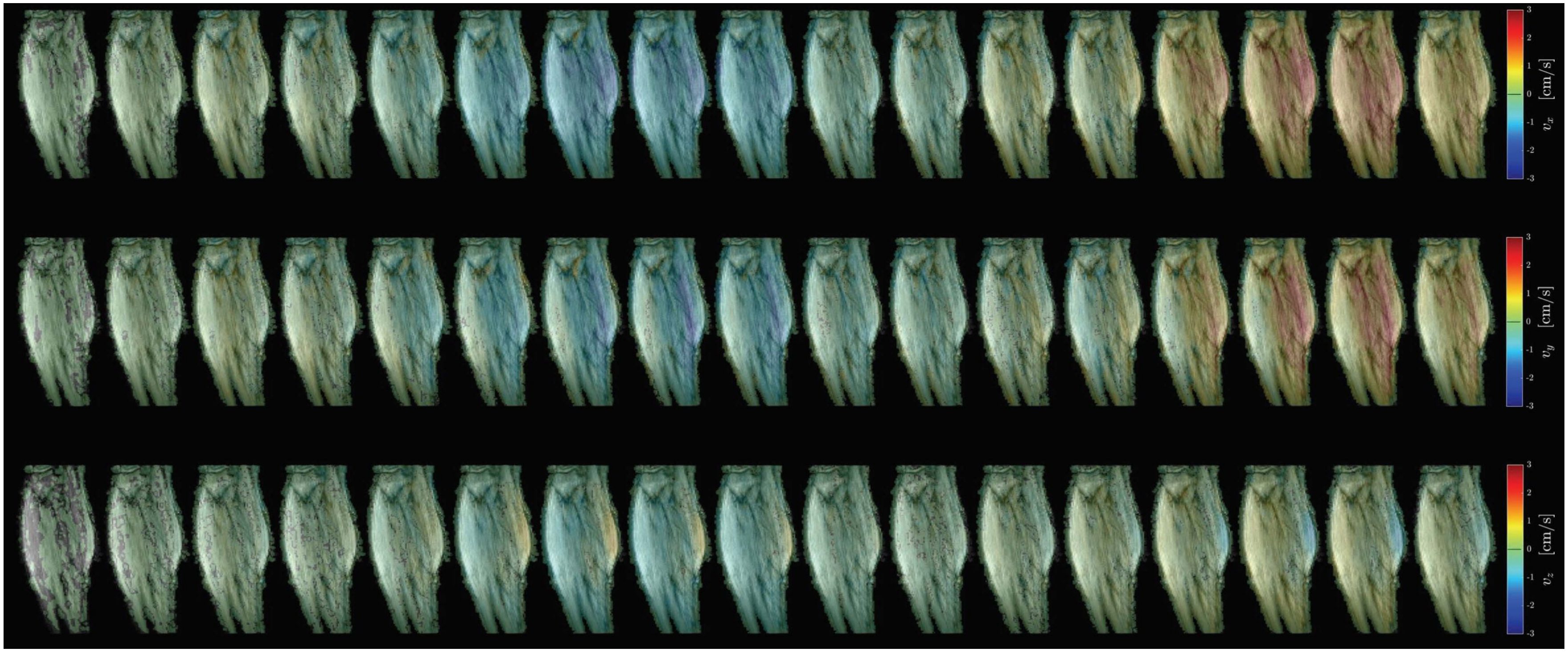

Supplementary Figure 4a. Velocity maps computed for a senior subject at 60%MVC. Velocity maps are shown through the first 2.3 seconds of the 3 second temporal cycle of the isometric contraction (17 temporal frames). The transition from contraction to relaxation part of the isometric contraction occurs around temporal frame 13. The change in velocities from negative (during the contraction) to positive (during relaxation) can be visually appreciated. The velocity maps have the highest values along the *y*-axis (v_y) (longitudinal muscle axis); the muscle fiber also primarily runs in craniocaudal direction (middle row). The intermediate values are seen along the *x*-direction (v_x) which is in the imaging plane, the plane of the muscle fibers (top row). The smallest values are seen in the *z*-directions (v_z) which is the out-of-plane direction (bottom row). In addition to the temporal variations, the decrease in velocity with %MVC (comparing S3a to S3b to S3c) is also visually evident.

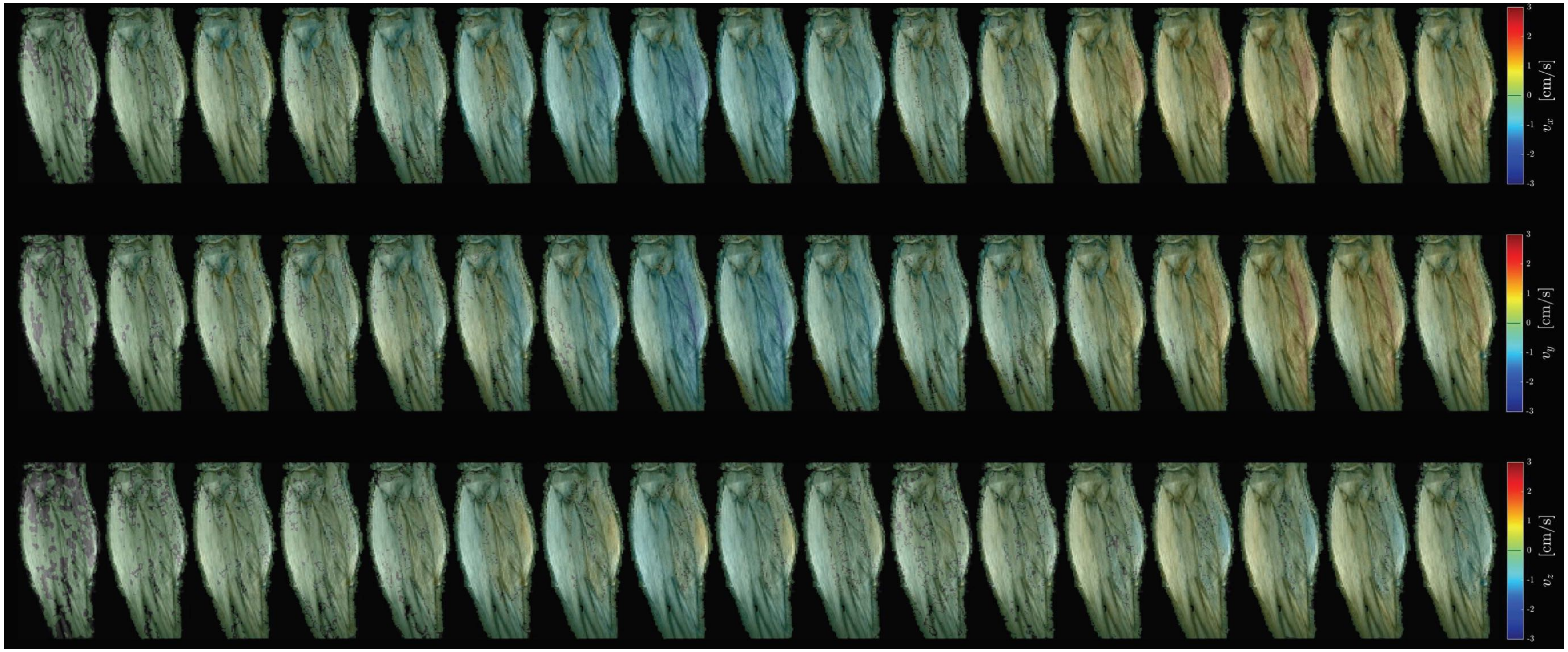

Supplementary Figure 4b. Velocity maps computed for a senior subject at 40%MVC.

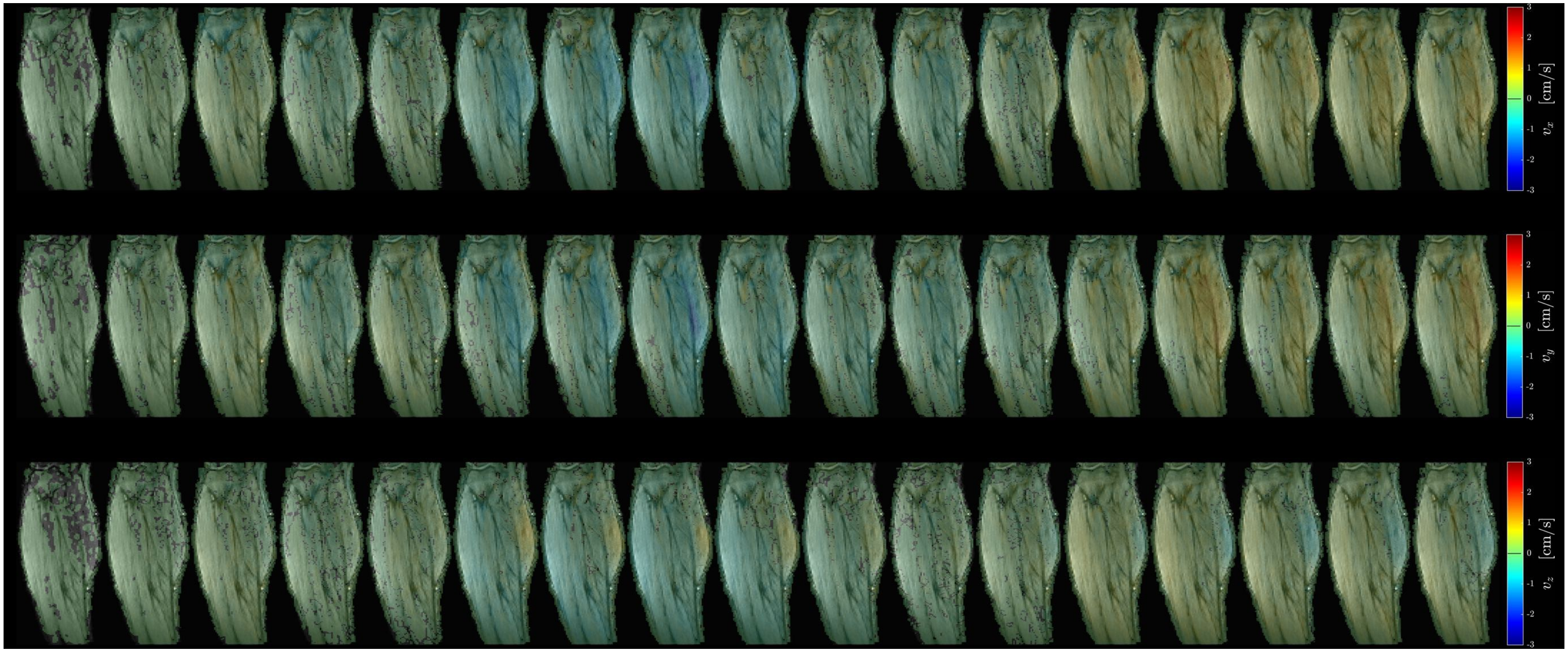

Supplementary Figure 4c. Velocity maps computed for a senior subject at 30%MVC.

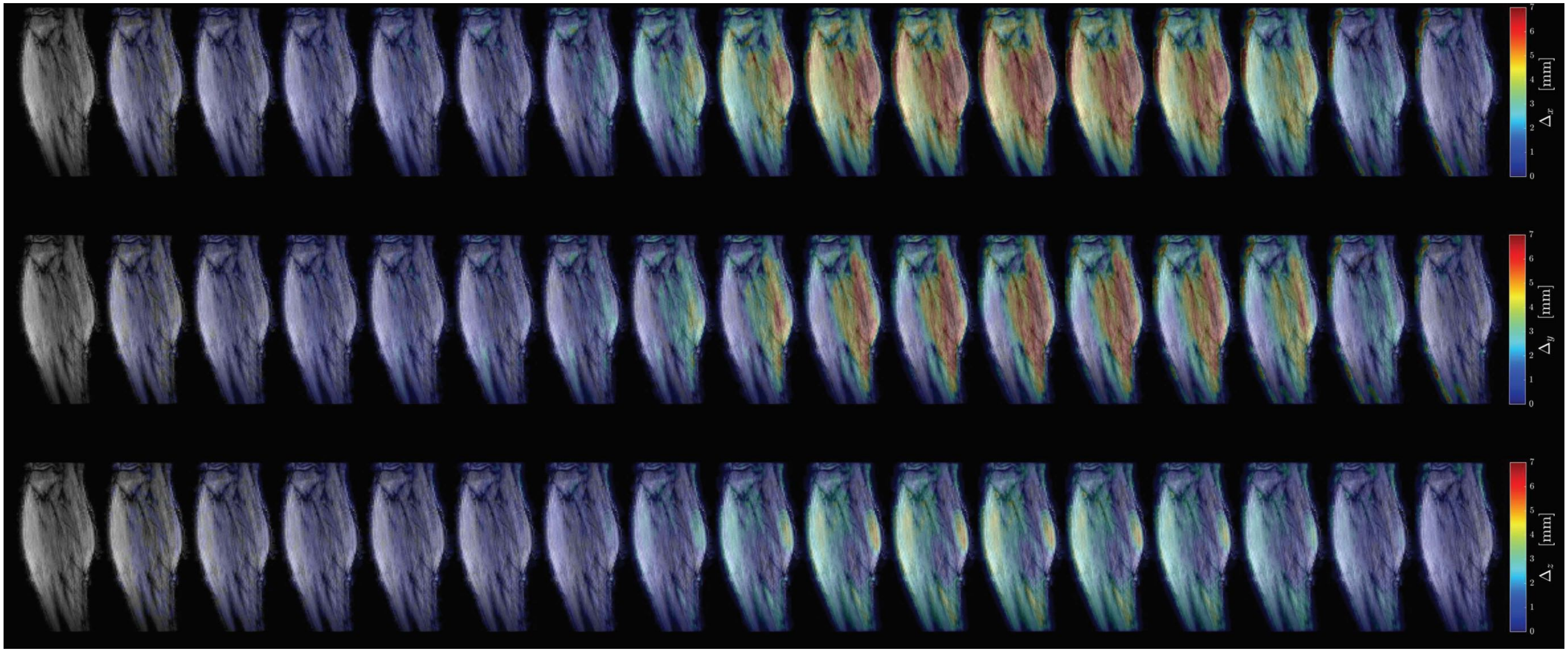

Supplementary Figure 4d. Displacement maps computed for a senior subject at 60%MVC. Displacement maps are shown through the first 2.3 seconds of the 3 second temporal cycle of the isometric contraction (17 temporal frames). The maximum displacement occurs at the peak of the force curve (frame 13). The displacement maps have the highest values along the y-axis (Δ_y) in conformance with the direction of the largest velocity values. The intermediate displacement values are seen along the *x*-direction (Δ_x) which is in the imaging plane, the plane of the muscle fibers (top row). The smallest displacements are seen in the *z*-direction (Δ_z) which is the out-of-plane direction (bottom row). Displacements, similar to velocity, decrease as the MVC decreases from 60% to 30% MVC.

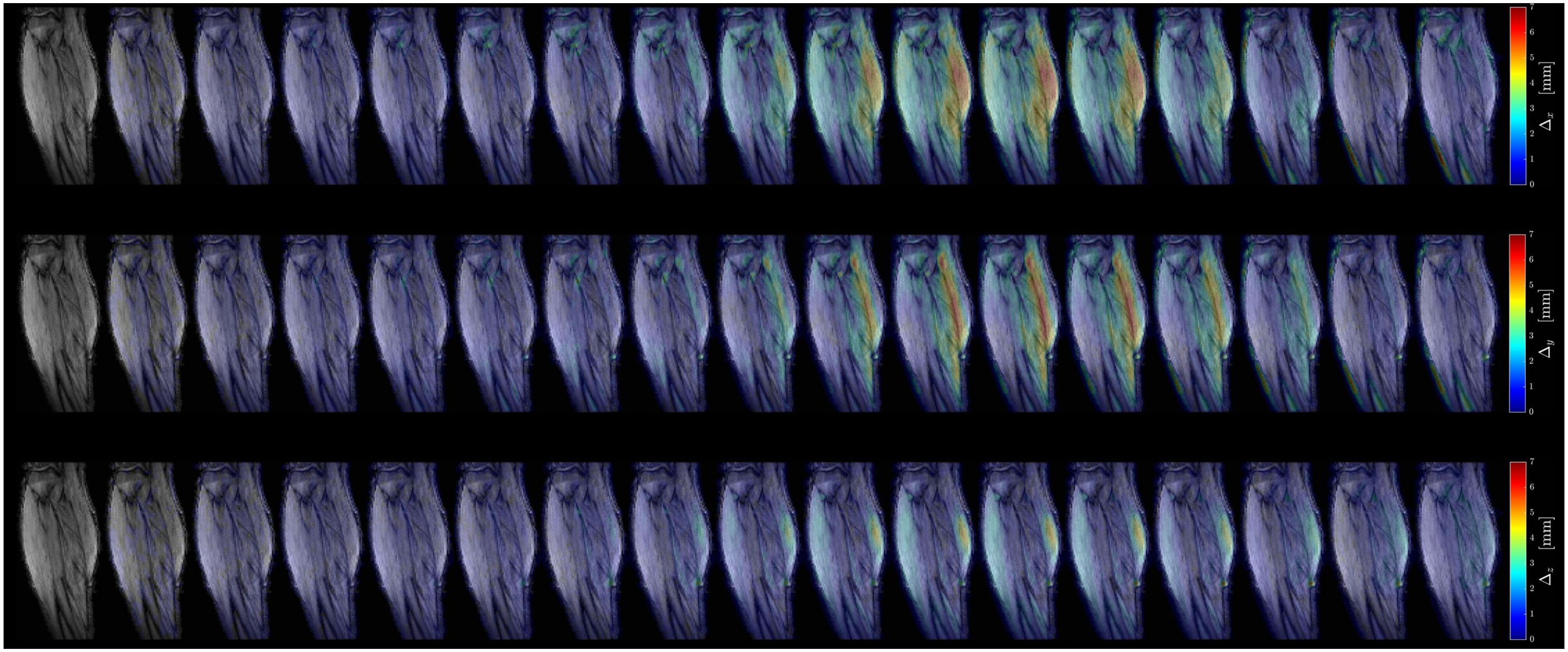

Supplementary Figure 4e. Displacement maps computed for a senior subject at 40%MVC.

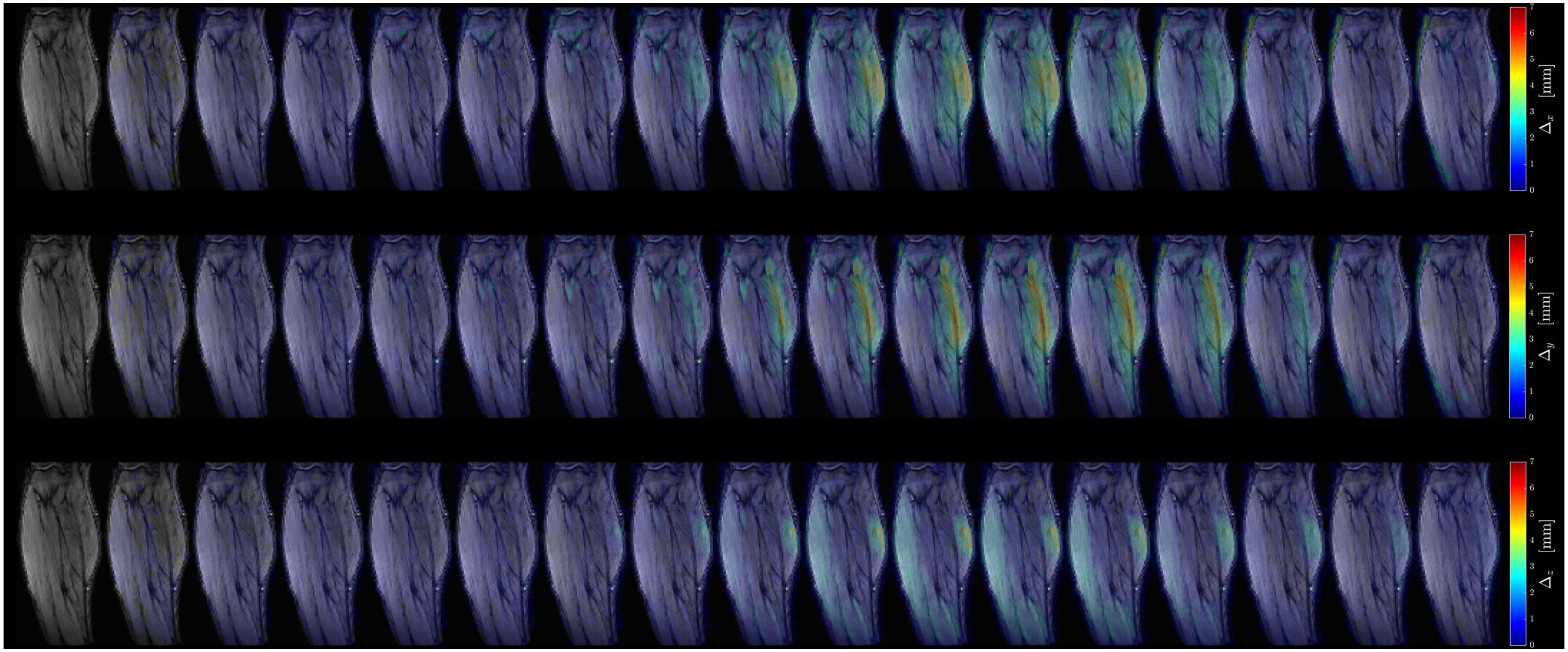

Supplementary Figure 4f. Displacement maps computed for a senior subject at 30%MVC.

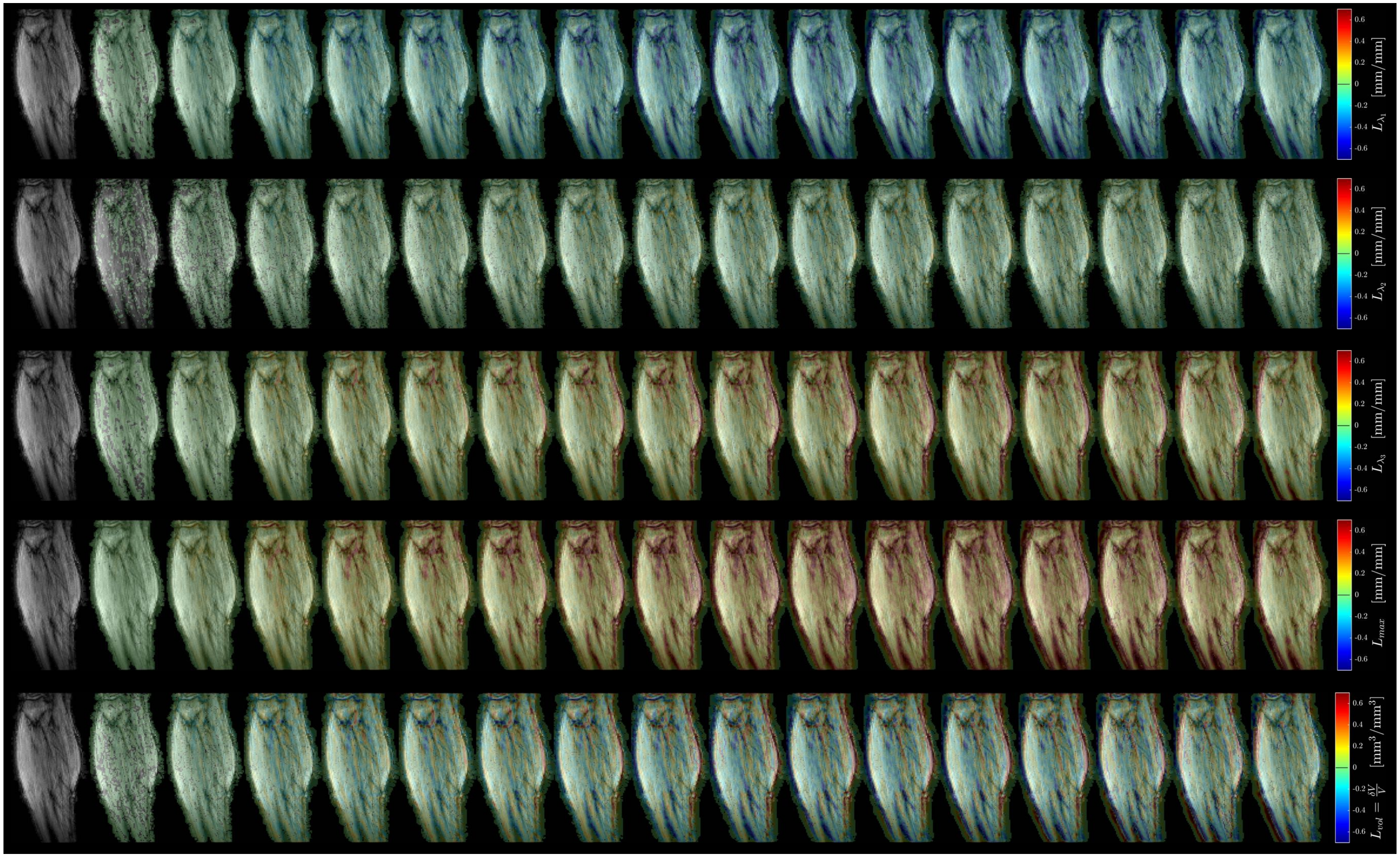

Supplementary Figure 4g. Strain maps computed for a senior subject at 60%MVC. The top three rows are the eigenvalues of the strain tensor arranged in increasing values from first to the third row. The negative eigenvalue (E_{λ_1}) is shown in the top row and during the contraction phase the negative eigenvalue is approximately along the muscle fiber and denoted as E_{fiber}. The second row shows the maps of the second eigenvalue (E_{λ_2}), it has the smallest absolute value and the direction of the strain is orthogonal to the imaging plane: $E_{\text{out-plane.}}$ The third row is the map of the third eigenvalue (E_{λ_3}), it is positive, and during the contraction phase, the direction of the strain is in the fiber cross-section in the imaging plane: $E_{\text{in-plane}}$. The last two rows are the two invariants of the strain tensor: maximum shear strain: E_{max} and volumetric strain: E_{vol}. The decrease in the values of the strain components as %MVC decreases can be visually appreciated.

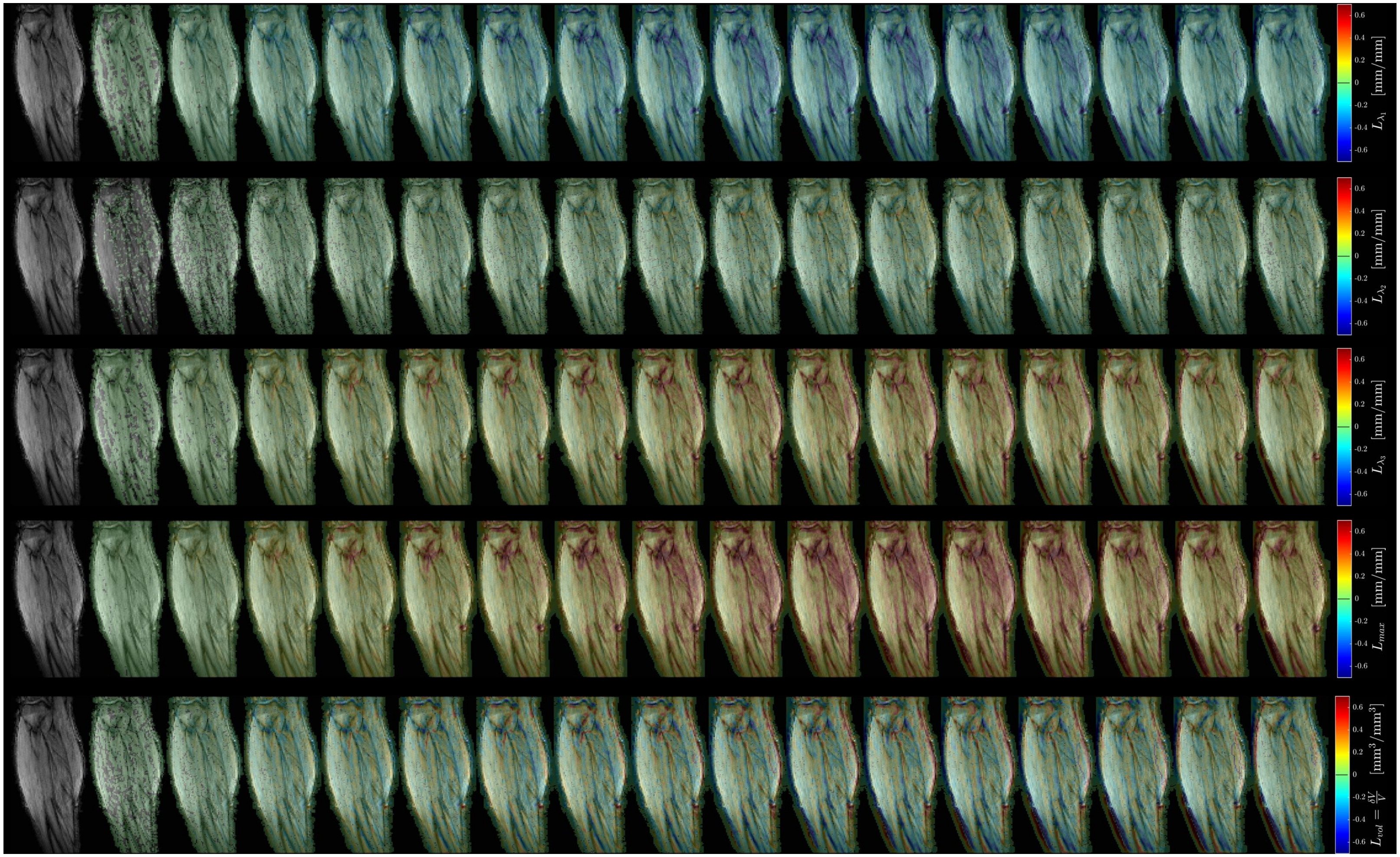

Supplementary Figure 4h. Strain maps computed for a senior subject at 40%MVC.

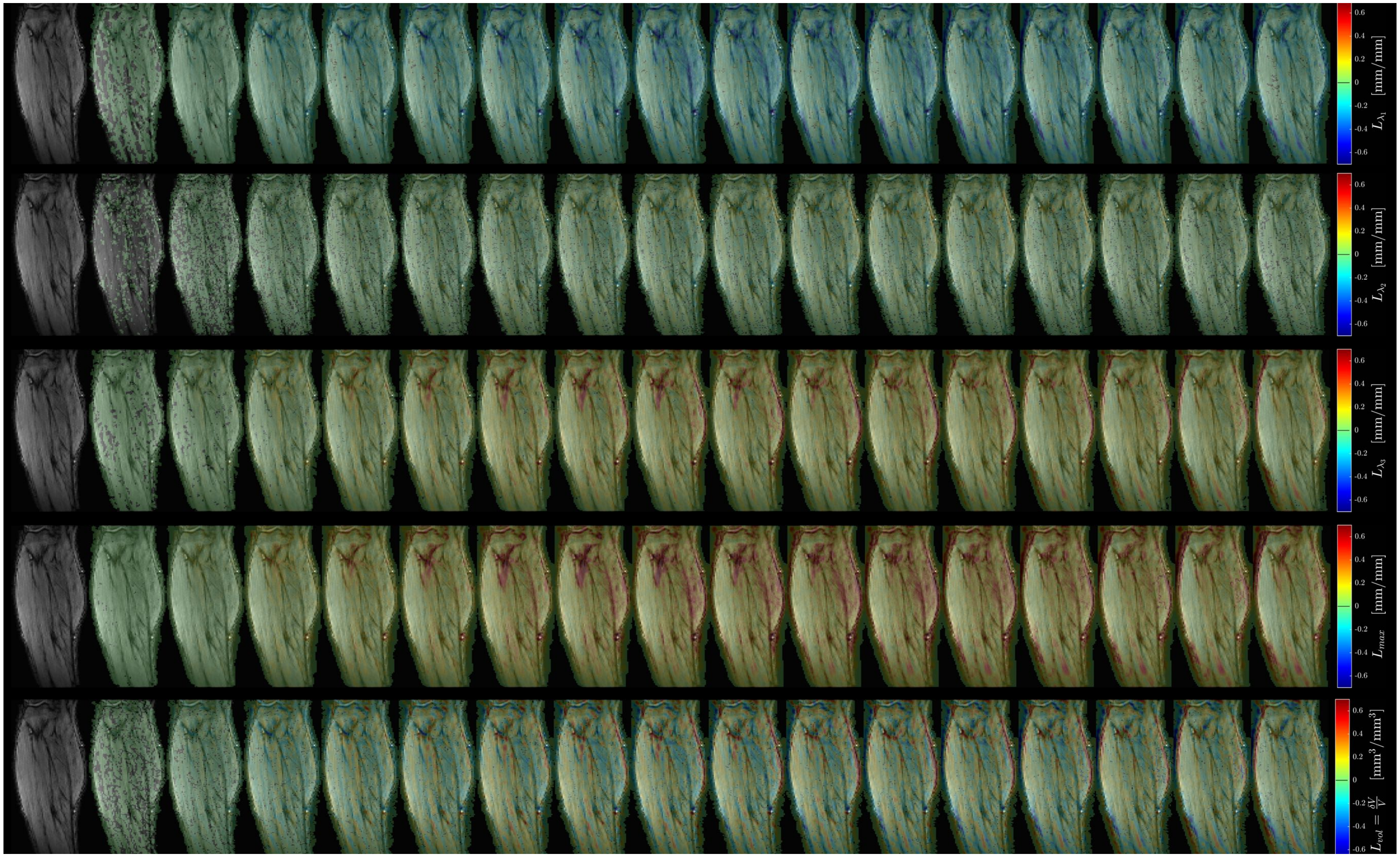

Supplementary Figure 4i. Strain maps computed for a senior subject at 30%MVC.

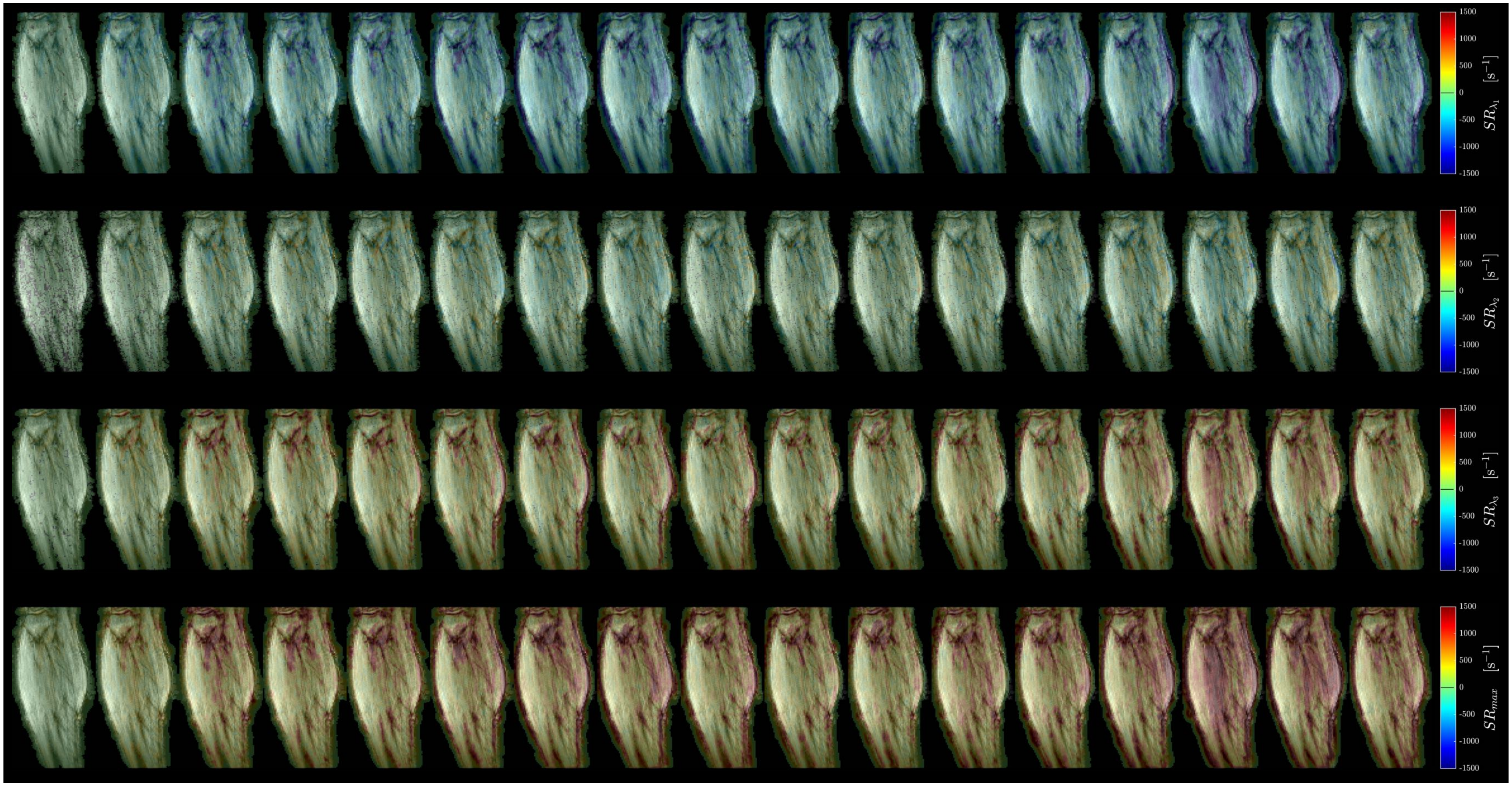

Supplementary Figure 4j. Strain rate maps computed for a senior subject at 60%MVC. The top three rows are the eigenvalues of the strain rate tensor arranged in increasing values from first to the third row. The negative eigenvalue (SR_{λ_1}) is shown in the top row and during the contraction phase the negative eigenvalue is approximately along the muscle fiber and denoted as $SR_{\text{fiber.}}$ The second row shows the maps of the second eigenvalue (SR_{λ_2}), it has the smallest absolute value and the direction of the strain is orthogonal to the imaging plane: $SR_{\text{out-plane.}}$ The third row is the map of the third eigenvalue (SR_{λ_3}), it is positive, and during the contraction phase, the direction of the strain is in the fiber cross-section in the imaging plane: $SR_{\text{in-plane.}}$ The last row is the map of the maximum shear strain: SR_{max}. The decrease in the values of the strain components as %MVC decreases can be visually appreciated.

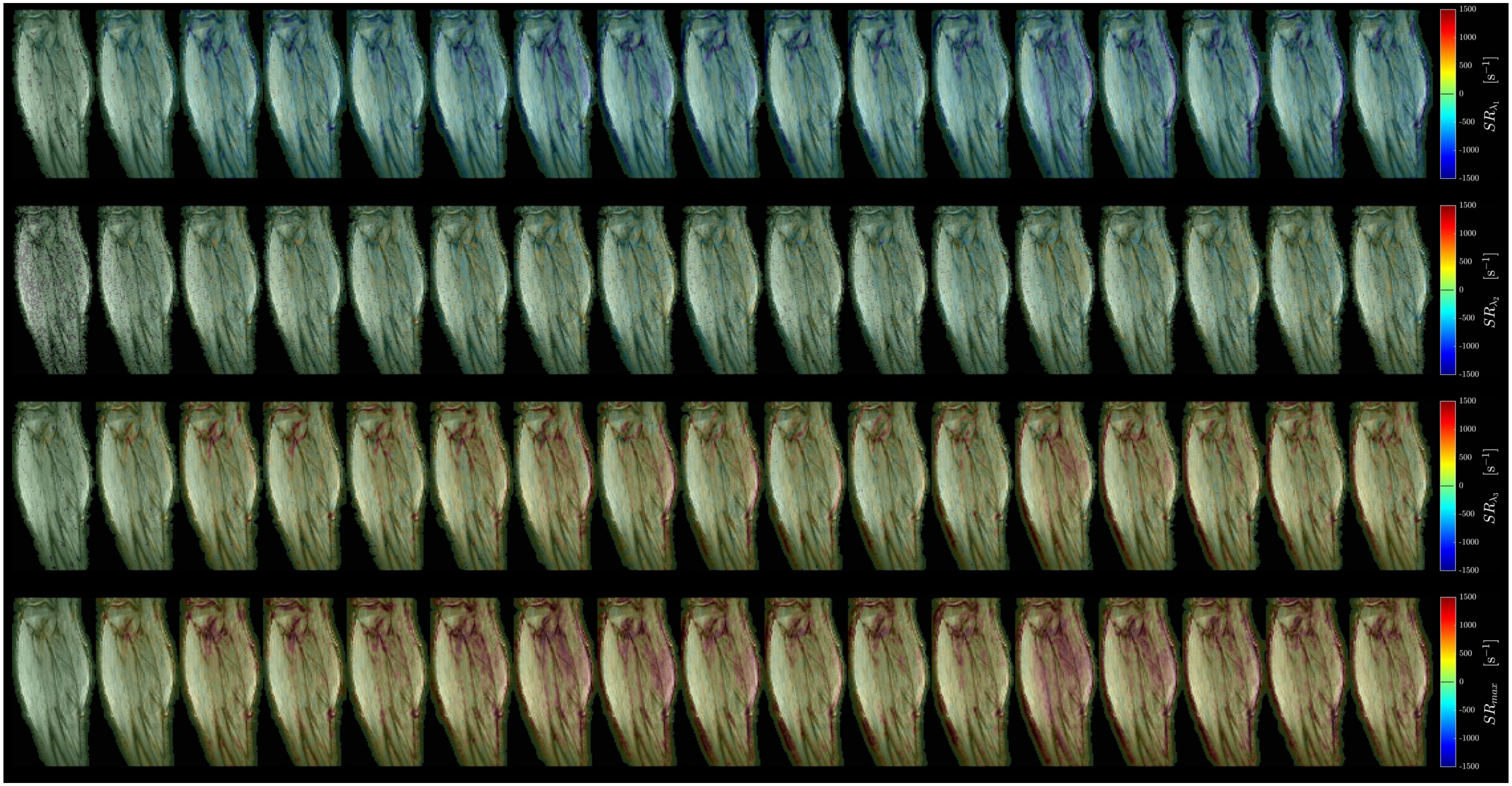

Supplementary Figure 4k. Strain rate maps computed for a senior subject at 40%MVC.

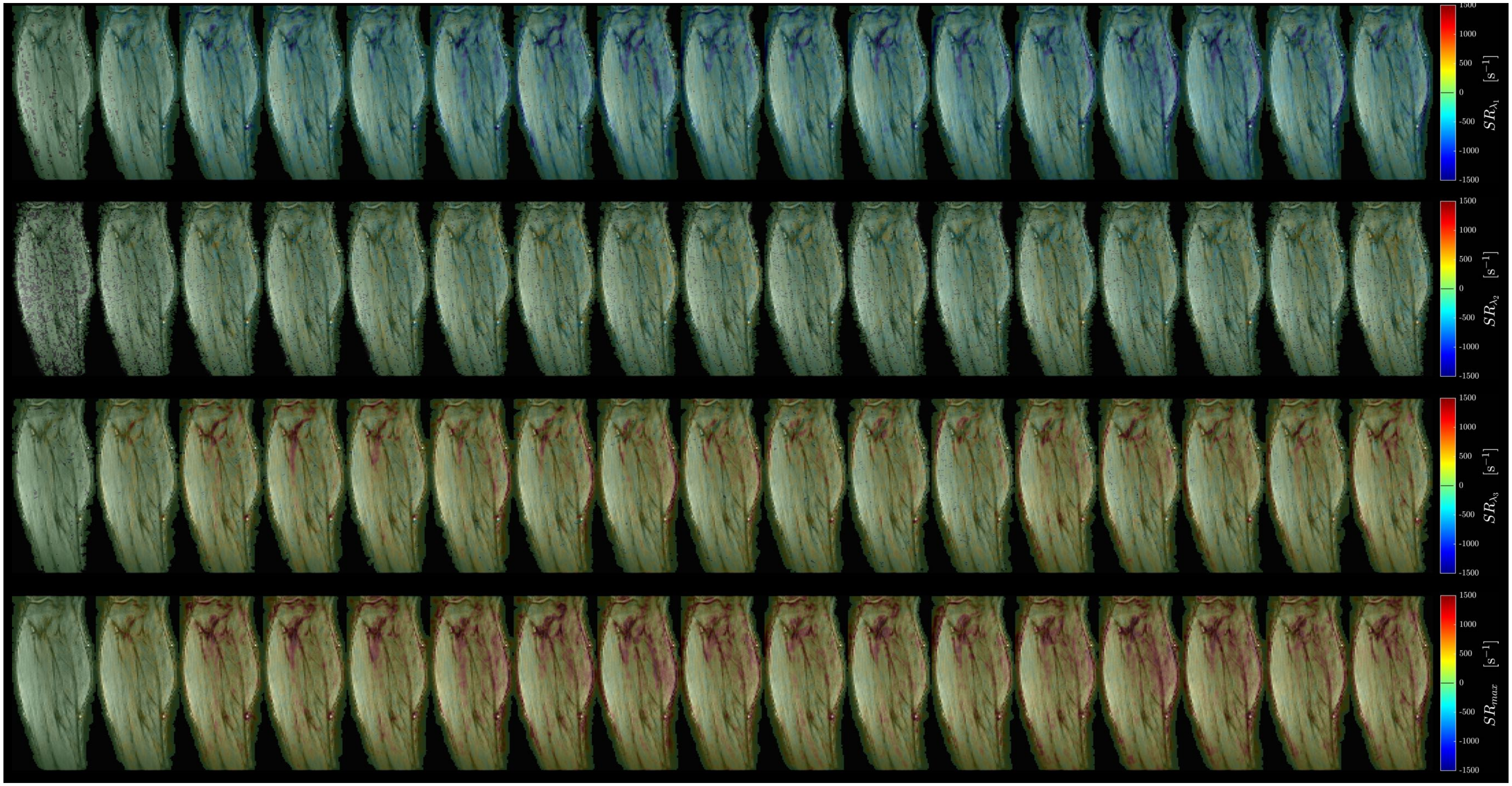

Supplementary Figure 4l. Strain rate maps computed for a senior subject at 30%MVC.
